# Facet‐specific Active Surface Regulation of Bi_*x*_MO_y_ (M=Mo, V, W) Nanosheets for Boosted Photocatalytic CO_2_ reduction

**DOI:** 10.1002/anie.202212355

**Published:** 2022-11-15

**Authors:** Yanzhao Zhang, Xing Zhi, Jeffrey R. Harmer, Haolan Xu, Kenneth Davey, Jingrun Ran, Shi‐Zhang Qiao

**Affiliations:** ^1^ Centre for Materials in Energy and Catalysis School of Chemical Engineering and Advanced Materials The University of Adelaide Adelaide SA 5005 Australia; ^2^ Centre for Advanced Imaging University of Queensland Brisbane 4072 Australia; ^3^ Future Industries Institute University of South Australia Mawson Lakes SA 5095 Australia

**Keywords:** Bi-Based Photocatalysts, CO_2_ Chemisorption and Activation, In Situ Spectroscopy, Photocatalytic CO_2_ Reduction, Specific Surface Regulation

## Abstract

Photocatalytic performance can be optimized via introduction of reactive sites. However, it is practically difficult to engineer these on specific photocatalyst surfaces, because of limited understanding of atomic‐level structure‐activity. Here we report a facile sonication‐assisted chemical reduction for specific facets regulation via oxygen deprivation on Bi‐based photocatalysts. The modified Bi_2_MoO_6_ nanosheets exhibit 61.5 and 12.4 μmol g^−1^ for CO and CH_4_ production respectively, ≈3 times greater than for pristine catalyst, together with excellent stability/reproducibility of ≈20 h. By combining advanced characterizations and simulation, we confirm the reaction mechanism on surface‐regulated photocatalysts, namely, induced defects on highly‐active surface accelerate charge separation/transfer and lower the energy barrier for surface CO_2_ adsorption/activation/reduction. Promisingly, this method appears generalizable to a wider range of materials.

## Graphical abstract

This work reports (010) specific surface‐regulated Bi_2_MoO_6_ nanosheets. The modified surface exhibits boosted CO_2_ photoreduction because of increased activation/adsorption of CO_2_ molecules. A combination of advanced characterizations and theoretical simulation confirmed performance and mechanism.

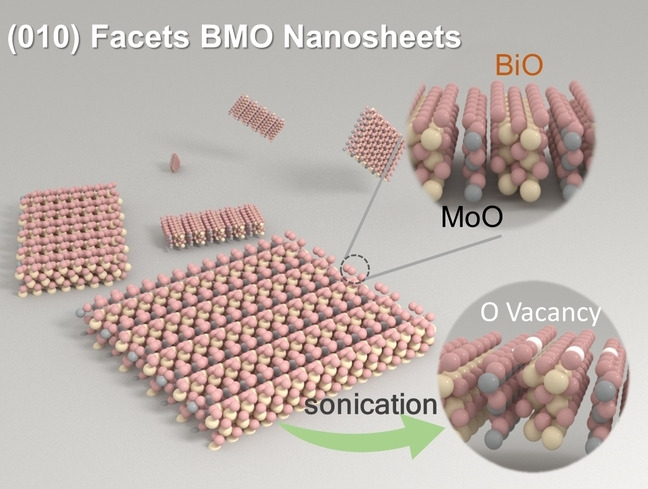

## Introduction

Photocatalytic carbon dioxide (CO_2_) reduction via semiconductor‐based catalysts is used for carbon fixation and green‐energy production.^[1]^ However, the thermodynamically stable CO_2_ molecule and passivated photocatalyst surfaces result in a significant barrier for CO_2_ adsorption/activation.^[2]^ Surface modification including, surface defects,^[3]^ single atoms^[4]^ and functional groups^[5]^ can boost CO_2_ molecule chemisorption and activation in photocatalytic CO_2_ reduction. These surface engineering changes can improve the active site and its coordination configuration on photocatalysts, boosting activity/selectivity for CO_2_ conversion.^[6]^ This is because, 1) the surface photogenerated charges are redistributed following surface regulated change, with electrons accumulating around active sites to facilitate reductive reactions[^6^, ^7^] and 2) surface active sites with optimized coordination structure boost reactant chemisorption/activation and reduce the energy barrier for CO_2_ reduction.^[8]^

Despite these advantages with surface engineering, it remains practically difficult to control surface defect concentration/distribution at high temperature and/or strong reducing conditions, because a bulk defect can readily be generated that will act as the recombination centre to reduce charge separation efficiency.^[9]^ Additionally, the surface vacancy can be healed by oxygen atoms released from CO_2_ molecules following the dissociation of C−O bonds.^[10]^ These might lose affinity to CO_2_ molecules and reduce activity/stability of the photocatalyst.^[11]^ The introduction of metal atoms, especially noble metals, is expensive and results in instability of photocatalysts. Moreover, it is complex to graft functional groups onto photocatalysts because introduced organic moieties are usually decomposed with illumination, and generate carbon contamination in CO_2_ conversion.[^5b^, ^12^] Therefore, an improved understanding is needed to better control processes for stable and well‐defined surface chemistry and boosted photocatalytic performance.

Active surface regulation of crystals can be rationally used to promote performance.[^8a^, ^13^] Controlled change can be made via formation of defects under strongly‐reductive chemicals (hydrazine, sodium borohydride and hydrogen gas)^[14]^ and high temperature/pressure.^[15]^ However, these require toxic/expensive chemicals and high energy input, leading to high emissions. Sonication is an attractive alternative because it can be used with usual reductive chemicals under ambient condition to generate cavitation, resulting in local‐area high temperature/pressure.^[16]^ Reactions therefore that are difficult at room temperature/atmosphere pressure can be got using reductive chemicals with sonication treatment.

Here we report active surface regulation of the (010) facets oxygen vacancies on a series of nanostructured photocatalysts, including Bi_2_MoO_6_, BiVO_4_ and BiWO_4_ via sonication‐assisted chemical reduction. The regulated surface is determined from combined advanced characterizations including X‐ray photoelectron spectroscopy (XPS), synchrotron‐based X‐ray absorption near‐edge structure (XANES) and extended X‐ray absorption fine structure (EXAFS) spectroscopy. Such proposed system exhibited three times photocatalytic CO_2_ reduction performance together with excellent stability and reproducibility, compared with unmodified photocatalyst. Boosted performance originates from the modified surface that promotes chemisorption/activation of CO_2_ molecules, reduced energy barrier in pathway and charge separation. The pathway for CO_2_ reduction and rate‐limiting steps are studied. The topmost surface of photocatalysts is impacted, whilst the crystal structure in the bulk is unaffected. This increases the electron‐hole dissociation/transfer and CO_2_ adsorption/activation on the surface, and reduces electron‐hole recombination in the bulk, as was evidenced by in situ spectroscopy, photoluminescence spectroscopy and theoretical computations.

## Results and Discussion

**Figure 1:**
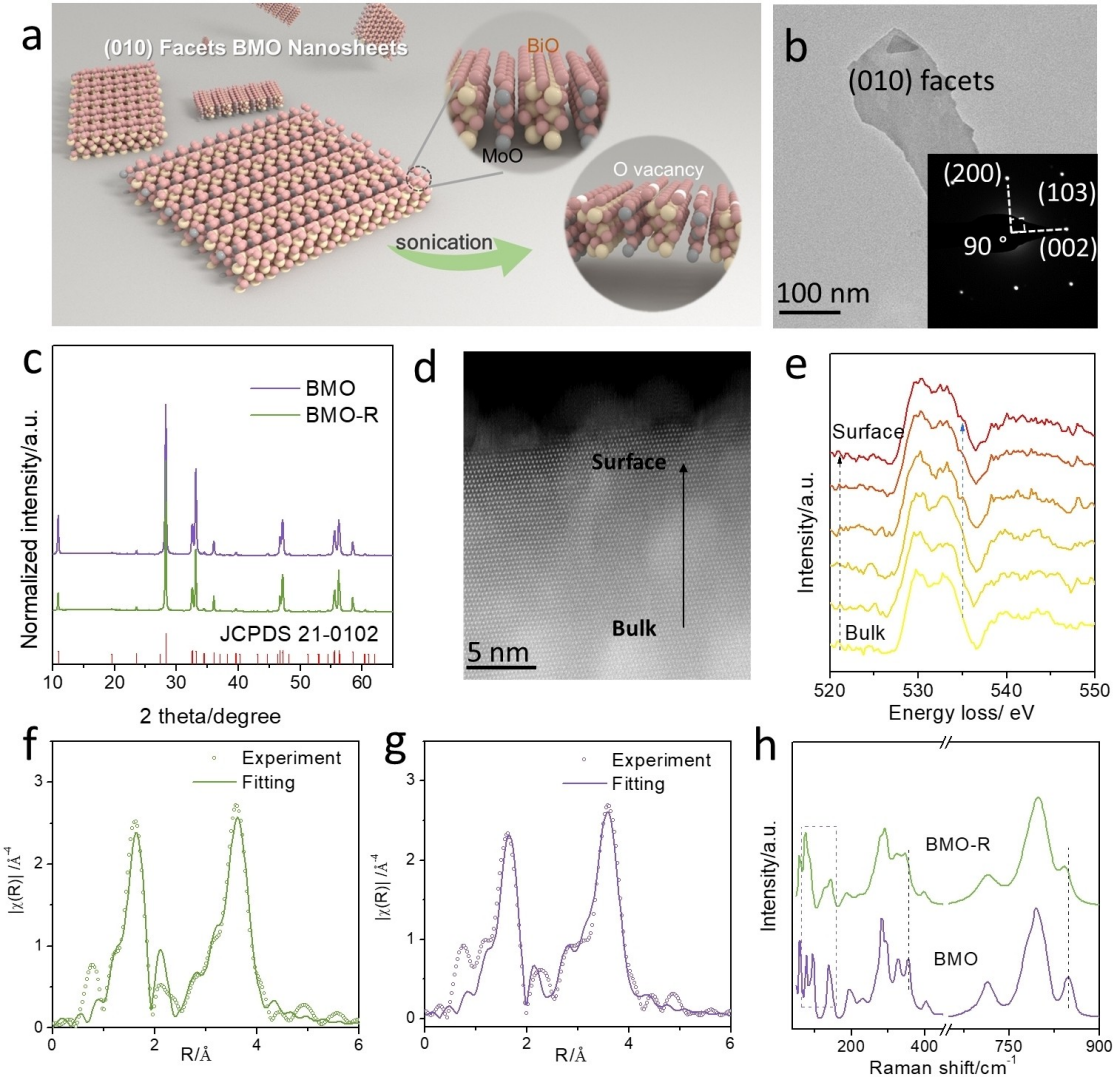
a) Scheme for BMO nanosheet crystal structure. Bismuth, molybdenum, oxygen and oxygen vacancy are denoted as balls, respectively, yellow, grey, red and white; b) TEM image and SAED, c) XRD patterns for BMO and BMO‐R; d) STEM image and e) series of O K‐edge EELS spectra from bulk to surface of BMO‐R; Bi L_3_ edge XAS experiment and fitted data for f) BMO‐R and g) BMO; h) Raman spectra for BMO and BMO‐R.

Bi_2_MoO_6_ nanosheet (BMO) was synthesized via hydrothermal reaction. The as‐synthesized BMO was cleaned (see details in Supporting Information) to remove organic surfactant. Surface‐regulated BMO (BMO‐R) via controlled sonication‐assisted chemical reduction in which BMO was dispersed in 80 mM Na_2_SO_3_ aqueous solution and subjected to sonication (Figure 1a). The sonication‐induced cavitation with local‐area high temperature/pressure, to significantly decrease the energy barrier to reduce BMO by Na_2_SO_3_ and significantly promote reduction of BMO by Na_2_SO_3_. This generates oxygen vacancies (V_O_) on BMO (010) facets as is demonstrated in the following reaction 1:

(1) $$\mathrm{Bi}{}_{2}\mathrm{MoO}{}_{6}+x\;\mathrm{SO}{}_{3}{}^{2-}\to \mathrm{Bi}{}_{2}\mathrm{MoO}{}_{6-x}+x\;\mathrm{SO}{}_{4}{}^{2-}+x\;V{}_{O}$$

Oxygen vacancies in BMO‐R were confirmed via electron paramagnetic resonance (EPR). As is shown in Figure S1, the stronger signal assigned to oxygen vacancy was observed from BMO‐R, not BMO. This implies that the metal ions are slightly reduced because of oxygen depletion. XPS and XANES were conducted to determine electronic states for BMO‐R. In Figure S2, the two peaks at 164.4 and 159.1 eV in XPS spectra are attributed to Bi 4f peaks of BMO. The two peaks at 235.5 and 232.4 eV are attributed to Mo 3d peaks of BMO.^[17]^ Both Mo 3d and Bi 4f peaks for BMO‐R exhibit a ≈0.2 eV shift toward lower binding energy, compared with BMO because of the oxygen loss. The depletion of oxygen is also confirmed from O 1s XPS spectra (Figure S3).The peak at 531.6 eV is attributed to the change of oxygen caused by oxygen deprivation and lattice distortion.^[18]^ BMO‐R exhibited a significantly greater proportion of this peak than BMO, indicating massive oxygen vacancies formed in BMO‐R. The adsorbed OH species is observed from 533.4 eV on BMO‐R (nothing for BMO), evidencing boosted H_2_O adsorption/activation on BMO‐R.^[19]^ The better affinity toward water on BMO‐R is favourable for water oxidation and proton generation for CO_2_ reduction. Additionally, the XANES spectra (Figure S4) for the Mo L‐edge for BMO‐R exhibit a “slight” reduction in the chemical state for Mo, compared with that for BMO. Such change in the chemical state of Mo is consistent with the findings in the XPS, confirming the formation of oxygen vacancies on BMO‐R following active surface‐regulated sonication. In contrast, where only sonication treatment or Na_2_SO_3_ aqueous solution is used, the reduction of BMO and generation of surface oxygen vacancy do not meaningfully occur. This was confirmed by high‐resolution XPS (Figure S5 and S6) for BMO with only Na_2_SO_3_ aqueous solution treatment (BMO‐Na_2_SO_3_), or sonication (BMO‐sonication).

The existence of abundant oxygen vacancies on BMO‐R surface induces relaxation and rearrangement of surface atoms to form a structure different to the bulk. Further, advanced characterizations, including atomic resolution high‐angle annular dark field scanning transmission electron microscopy (HAADF‐STEM), were employed to assess the surface chemical structure of BMO‐R. As is shown in Figure 1b and Figure S7, these two samples exhibit similar, sheet‐like shapes. The as‐synthesized BMO nanosheets exhibited a mean thickness 10.9 nm (Figure S8). Following sonication‐assisted chemical reduction, BMO‐R exhibited reduced dimension with mean thickness 10.7 nm (Figure S9). These findings evidence that sonication did not significantly affect morphology of BMO. The crystal structure of BMO and BMO‐R was determined via X‐ray diffraction (XRD). As is seen in Figure 1c, all diffraction peaks for BMO and BMO‐R are attributed to orthorhombic‐structured Bi_2_MoO_6_ (JCPDS No. 21‐0102), denoting that sonication‐assisted chemical reduction did not meaningfully alter the crystal structure of BMO‐R. The selected area electron diffraction (SAED) pattern for BMO‐R is shown in Figure 1b inset. It can be indexed into diffraction spots along the [010] zone axis, confirming the single‐crystal structure of BMO‐R. Additionally, the atomic‐resolution HAADF‐STEM image of BMO‐R (Figure S10) demonstrated two lattice spacing values of 0.28 nm, with an angle of 90°, that is attributed to, respectively, (200) and (002) facets. It is concluded therefore that the exposed surface is (010) facets of BMO. Energy dispersive X‐ray (EDX) elemental mapping confirmed the elemental composition and distribution of the BMO‐R (Figure S11).

To assess the top surface of BMO‐R, combined atomic‐resolution HAADF‐STEM, local‐area electron energy loss spectroscopy (EELS), synchrotron‐based XANES/EXAFS and Raman spectroscopy were conducted. The atomic‐resolution HAADF‐STEM image of side‐view of BMO‐R (Figure 1d) evidences the regulated surface atoms on the topmost (010) facets. In contrast, the (010) facets for bulk BMO‐R exhibit an ordered and aligned structure. Additionally, EELS was collected to reveal the local oxidation state and coordination information. As the EELS area changed from the bulk to the surface of BMO (Figure 1e), the peak at ≈534 eV for the O K edges shows an apparent increase, evidencing oxygen deprivation on the topmost (010) facets of BMO‐R.^[20]^ XANES and EXAFS spectra for BMO and BMO‐R were determined to assess the chemical environment of BMO‐R. As is presented in Figure S12, the Bi L_3_‐edge XANES spectra on the BMO‐R and its corresponding derivative shift slightly to a lower direction compared with that for BMO, which confirms that the Bi oxidation state in BMO‐R is reduced. The slight shift was affected by the transmission mode for data collection, for the information from bulk crystals. The well‐coordinated lattice atoms reduce sensitivity. Fourier transform (FT) EXAFS corroborated the valence change. Both samples exhibited the dominant peak at the same radial distance of 1.6 Å that is assigned to the Bi−O bond. However, the intensity of the peak for BMO‐R weakens when compared with that for the pristine BMO, confirming the reduced Bi−O coordination number (Figure S13).^[21]^ Additionally, quantitative least‐squares EXAFS curve‐fitting demonstrated that Bi−O and Bi−Bi are two backscattering paths involved in BMO and BMO‐R. To get comparable results from two samples, the fitting analysis is based on the same backscattering paths. The coordination number for Bi−O decreased from 5.96 to 5.60 following sonication (Figures 1f, g, S14, and S15; Table S1). The reduced coordination number is attributed to the loss of lattice oxygen atoms. It is concluded these findings confirm the impact of active surface regulation. Raman spectroscopy was used to reveal the structure information and oxidation state of BMO‐R. As is presented in Figure 1h, the stretching and deformation of the MoO_6_ octahedral unit are seen in the transitions ca. 845 and 402 cm^−1^, respectively. The redshift for these two bands in BMO‐R evidences the distortion of the MoO_6_ unit. The partial oxygen loss of Bi_2_O_2_ unit is demonstrated via the differences in Raman spectra range <200 cm^−1^.^[22]^

A similar surface regulation at (010) facets was apparent in the other Bi‐based materials, BiVO_4_ and Bi_2_WO_6_. This is because these have a similar, layered crystal structure and electronic transfer properties. Corresponding characterizations XRD, Raman and XPS were used to determine the partial reduction and surface transformation of these (Figures S16 and S17).

**Figure 2:**
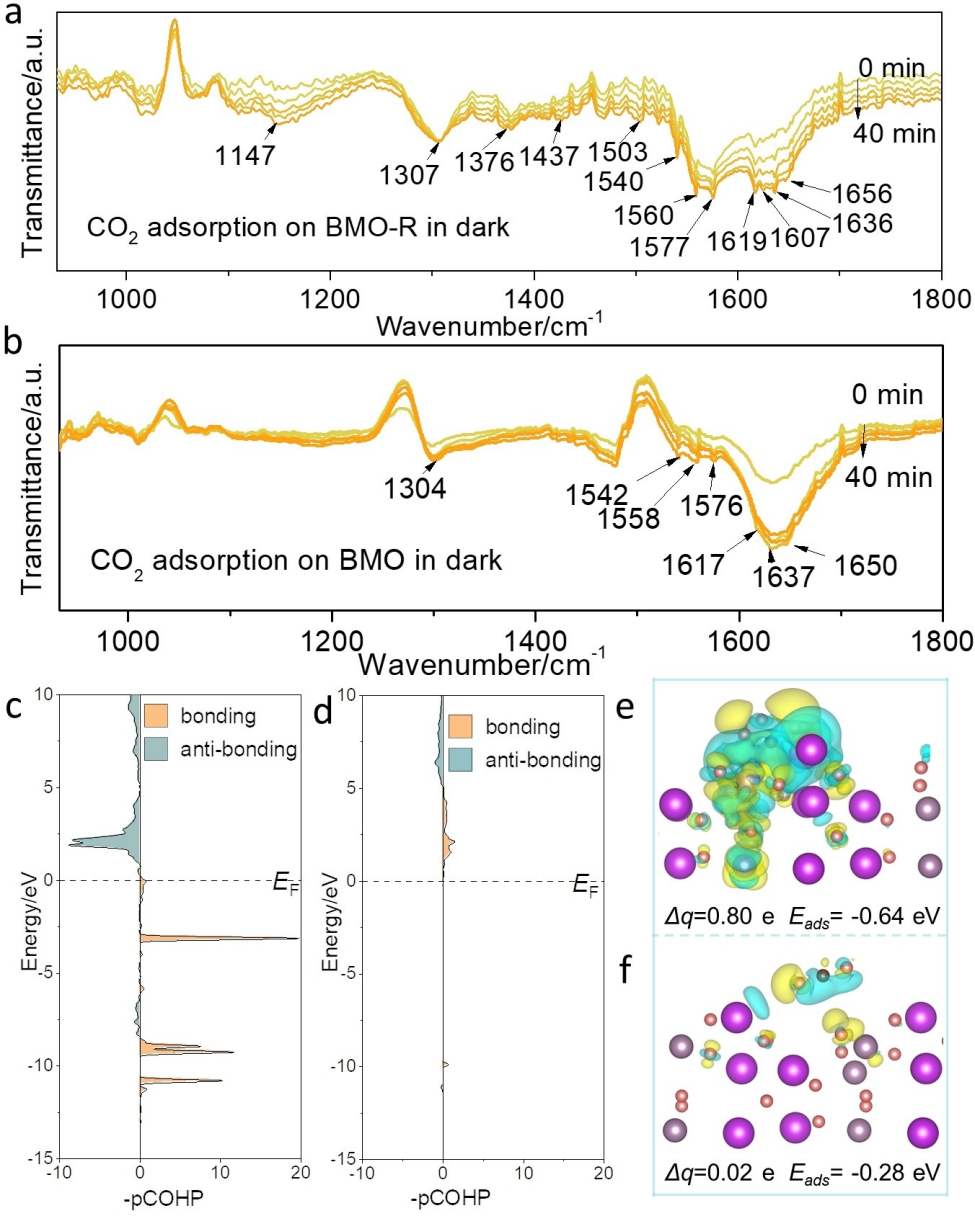
*In situ* DRIFTS test for CO_2_ and H_2_O interaction with a) BMO‐R and b) BMO in dark; Projected crystal orbital Hamilton population (pCOHP) between carbon atom in CO_2_ and Mo active site on c) BMO‐R and d) BMO; Charge difference distributions for e) BMO‐R and f) BMO following CO_2_ adsorption (charge depletion is in yellow and accumulation in blue, positive values for Δ*q* indicate electron accumulation on CO_2_ *E*_ads_ is CO_2_ adsorption energy on surface). Isosurfaces are 0.003 e Å^−3^. Oxygen, carbon, bismuth and molybdenum atoms are denoted as balls, respectively red, brown, purple and grey.

Compared with reported surface engineering, the as‐synthesized BMO‐R possesses a distinctive surface structure, distorted metal centre and coordination environment. The actively‐regulated surface boosts activity in photocatalysis. Because CO_2_ adsorption and activation on the surface is a prerequisite for photocatalytic CO_2_ reduction, in situ diffuse reflectance infrared Fourier transform spectroscopy (DRIFTS) in dark was carried out on BMO‐R, Figure 2a. Some intermediates are typical species for the products. The peaks ca. 1656 and 1607 cm^−1^ are attributed to ⋅CO_2_^−^, an important indicator for CH_4_ generation.^[23]^ The rising peaks at 1577, 1560, 1503 and 1307 cm^−1^ with increasing time are from the characteristic vibrations of monodentate carbonates (m‐CO_3_^2−^). The formation of bidentate carbonate species (b‐CO_3_^2−^) is evidenced by the peaks at 1540, 1376 and 1147 cm^−1^. The adsorption of H_2_O and CO_2_ is inferred from HCO_3_^2−^ (1437 and 1636 cm^−1^) and H_2_O vibrations (1619 cm^−1^).^[9b]^ The high concentration of protonation of carbonate species is significant as these are critical intermediates in CO production. Compared with the CO_2_ adsorption spectrum for BMO‐R, the detected intermediates are significantly less than on BMO (Figure 2b). The same peaks at 1650, 1637, 1617, 1576, 1558, 1542, 1437 and 1304 cm^−1^ are attributed to the intermediates, CO_2_^−^, HCO_3_^2−^, H_2_O, m‐CO_3_^2−^ and b‐CO_3_^2−^.[^6^, ^14b^] However, BMO mainly produces m‐CO_3_^2−^ rather than b‐CO_3_^2−^ which are the robust sites required for CO_2_ adsorption. Therefore BMO‐R exhibits significantly better CO_2_ adsorption/activation ability in CO_2_ photoreduction than BMO.

Theoretical computations were used to determine the interaction between CO_2_ and surface of BMO‐R. It is seen in Figure 2c, the majority of valence band for BMO‐R (below Fermi level, *E*_F_) are on the bonding orbital, whilst the antibonding orbital population mainly distributes in the conduction band, above *E*_F_. However, almost all the bonding orbital population (Figure 2d) for BMO distributes above *E*_F_, which confirms the weak interaction between CO_2_ and Mo active site. The projected crystal orbital Hamilton population (pCOHP) for CO_2_ adsorbed on BMO surface is −0.05, whilst that for BMO‐R is −3.41. To determine CO_2_ adsorption behaviour on different surfaces, the adsorption energy, charge difference and Bader charge analysis based on DFT computation for BMO and BMO‐R were carried out (Figures 2e and f). The Mo atom around the distortion centre caused by the oxygen deprivation was assessed. The CO_2_ adsorption energies on the two models exhibit a significant difference with, respectively, −0.64 and −0.28 eV on BMO‐R and BMO. On the surface of BMO‐R, the C−O−C bond was bent (134.5°) and elongated (1.27 and 1.25 Å) because of the electron (0.8 e) transfer from the surface to CO_2_ molecule on the BMO‐R (Figure S18). Additionally, the lower adsorption position on BMO‐R evidences that the CO_2_ molecule is more readily captured by the surface. For the BMO, the C−O−C bond was not bent significantly (178.6°) and elongated (1.17 and 1.17 Å). The Bader charge analysis confirmed that few electrons (0.02 e) transfer from surface to CO_2_ molecule. It is concluded that these experimental and computational findings demonstrate the stronger adsorption of CO_2_ on BMO‐R than BMO.

**Figure 3:**
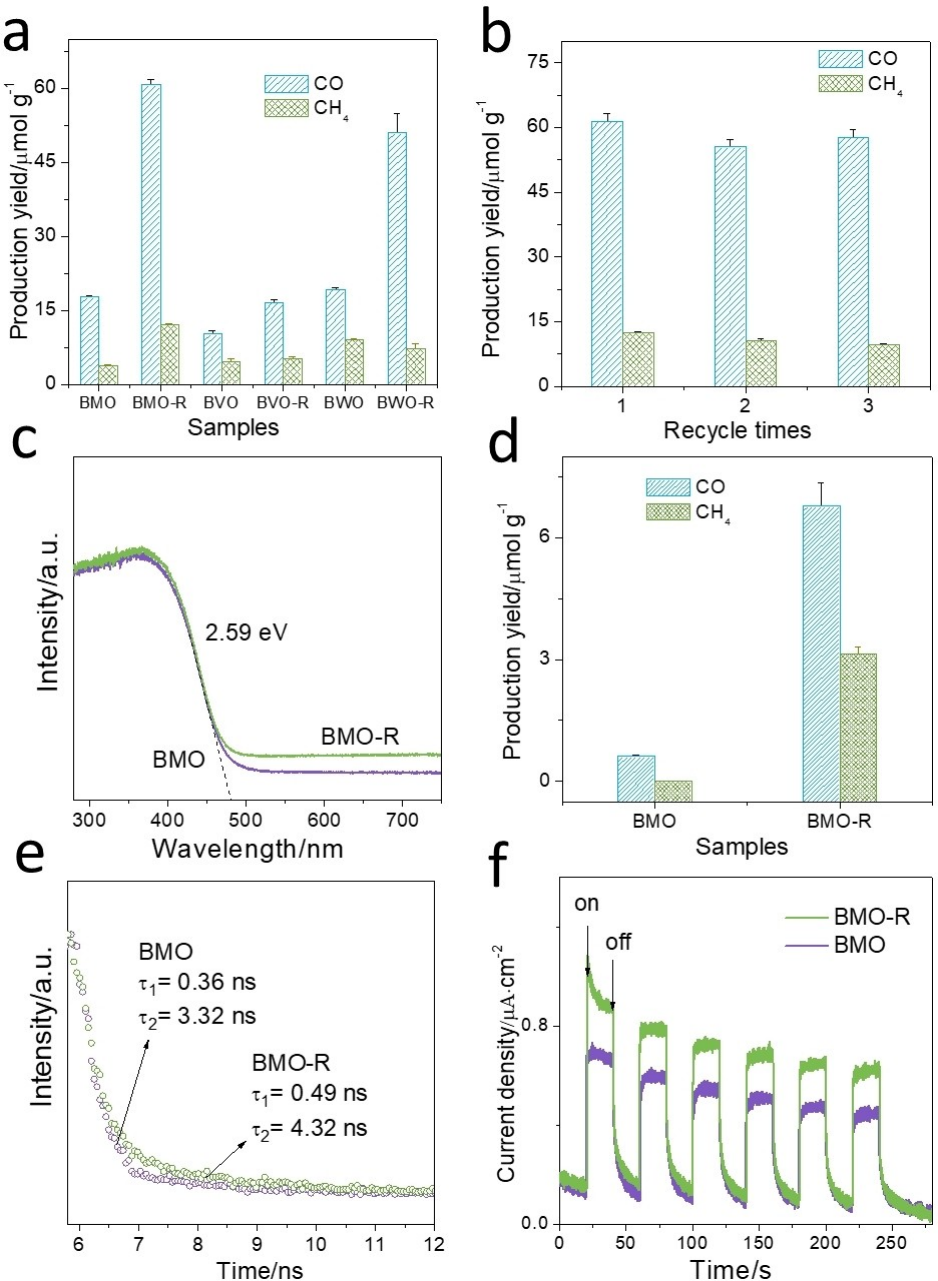
a) Photocatalytic CO_2_ reduction for BMO, BMO‐R, BVO, BVO‐R, BWO and BWO‐R under Xenon lamp illumination; b) Repeated photocatalytic CO_2_ reduction test for BMO‐R; c) UV/Vis diffuse reflectance spectroscopy and band gap for BMO and BMO‐R; d) CO_2_ photoreduction for BMO and BMO‐R under 540 nm LED illumination for 7 h; e) TSPL spectra for BMO and BMO‐R; f) Transient photocurrent density for BMO and BMO‐R in 0.5 M Na_2_SO_4_ aqueous solution.

As is shown in Figure 3a, BMO exhibited limited CO_2_ photoreduction with CO and CH_4_ production of, respectively, 17.7 and 3.3 μmol g^−1^. In comparison, BMO‐R exhibited CO and CH_4_ generation of, respectively, 60.7 and 12 μmol g^−1^, three times greater than for BMO. The performance for BVO‐R and BWO‐R were boosted following active surface regulation. The slight enhancement for BVO‐R is due to the poor reduction capability of photogenerated electrons. Samples were cleaned following synthesis to eliminate carbon impurities.^[12b]^ Compared with reported findings, BMO‐R, BWO‐R and BVO‐R show excellent CO_2_ photoreduction performance under similar conditions (Table S2).

Additionally, blank experiments were conducted under the same conditions and purged with ultra‐high‐purity Ar and not CO_2_. When the Ar was purged instead of CO_2_, negligible production was detected (Figure S19). Moreover, all the other blank experiments resulted in no products. This evidenced that CO and CH_4_ were generated from photocatalytic CO_2_ reduction. Stability was assessed with a three‐time recycle test of 7 h per cycle. No apparent deterioration in performance was found (Figure 3b). The increased stability likely comes from hindering healing oxygen vacancy by CO_2_ and H_2_O because of the distorted surface crystal and steric‐hindrance. XPS, TEM, XRD and SAED data (Figures S20 and S21) for BMO‐R following performance testing confirmed that the crystal structure and defects were stable, and therefore that the surface was not altered during CO_2_ photoreduction.

To determine the origin of boosted activity and reaction mechanism for photocatalytic CO_2_ reduction, UV/Vis diffuse reflectance spectroscopy was obtained. As is shown in Figure 3c, increased absorption in the range 480 to 700 nm is attributed to oxygen deprivation on BMO‐R (010) facets. However, compared with Figure S19, the performance enhancement is negligible (Figure 3d). However, no shift for the adsorption edge for BMO‐R (479 nm) evidences that its band gap (2.59 eV) is not changed. The positions of the valence band maximum (VBM) for BMO‐R and BMO were detected via XPS valence spectra, respectively, as 2.06 and 2.56 eV, (Figure S22). From the VBM and band gap, the conduction band minimum (CBM) for BMO‐R and BMO is estimated to be −0.53 and −0.03 eV. The CBM for BMO‐R exhibits upshift, which results in a greater reduction ability of photogenerated electrons and therefore facilitates CO_2_ photoreduction.

To determine photogenerated charge separation/transfer, steady‐state photoluminescence (PL) spectroscopy, transient‐state photoluminescence (TSPL) spectroscopy, transient photocurrent (TPC) density and electrochemical impedance spectroscopy (EIS) were obtained. The steady‐state PL intensity for BMO‐R is significantly less than that for BMO (Figure S23), evidencing that charge‐carrier recombination is suppressed. Following fitting of the TSPL curves for Figure 3e, lifetimes of charge carriers of *τ*_1_=0.49 ns and *τ*_2_=4.32 ns, for BMO‐R were greater in comparison with those for BMO of, respectively, 0.36 ns and 3.32 ns. BMO‐R exhibited a greater TPC density than BMO (Figure 3f). Because TPC density is significantly affected by surface reaction efficiency and electron transfer resistance, it is not correlated well the performance tests. This finding confirms the more efficient dissociation of light‐induced excitons. The EIS spectra (Figure S24) exhibited a smaller‐semicircle radius for the Nyquist plot, together with a decreased charge‐transfer resistance of *R*_ct_=3500 Ω for BMO‐R in contrast with that for BMO of 6750 Ω, confirming a greater charge carrier transfer rate in BMO‐R. The difference between the two evidences that the charge recombination was suppressed in BMO‐R because MoO_6−*x*_ sites act as traps for photogenerated electrons, therefore facilitating charge separation.^[24]^

**Figure 4:**
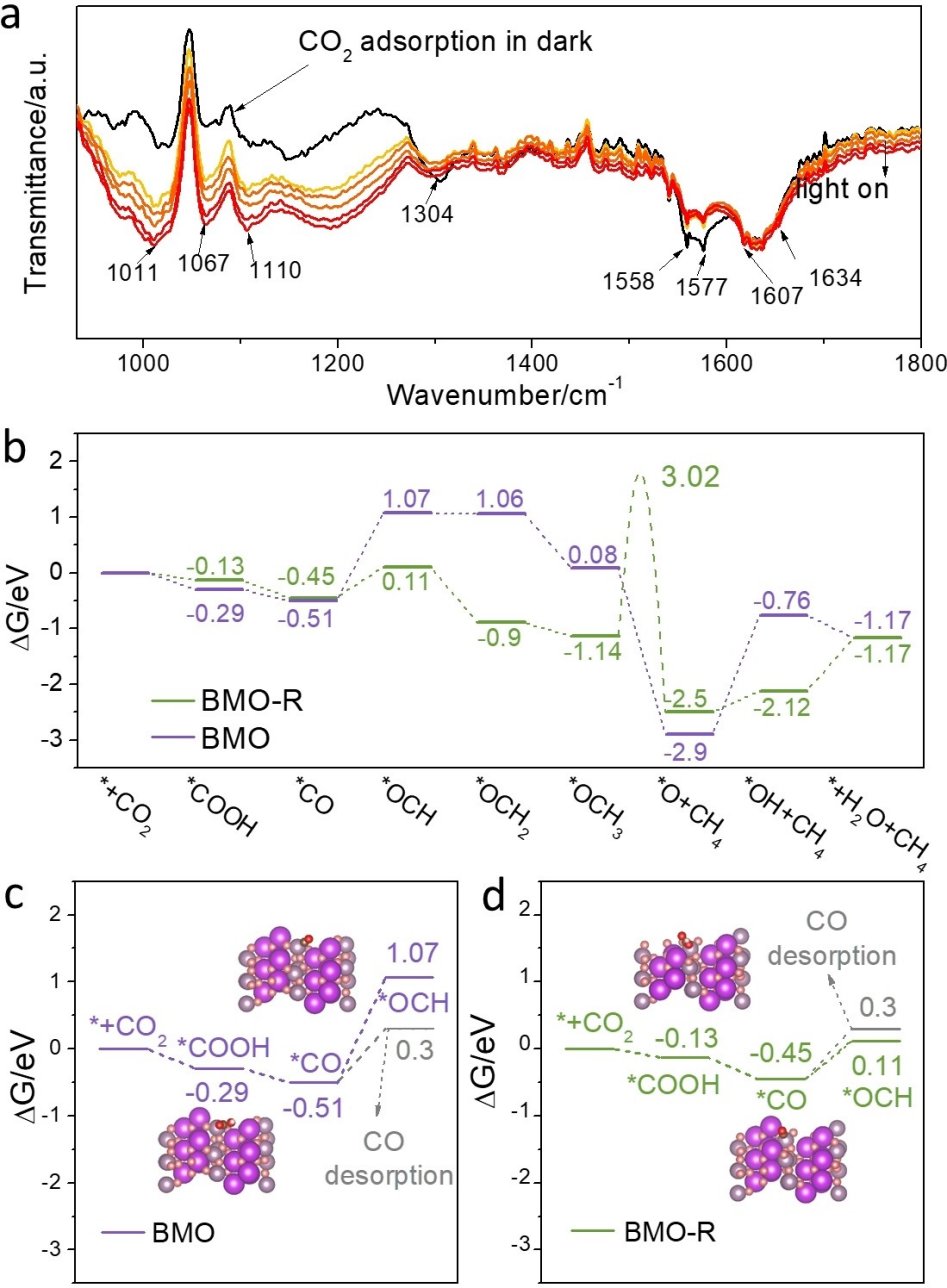
a) *In situ* DRIFTS test for CO_2_ and H_2_O interaction with BMO‐R under constant Xenon lamp illumination; b) Computed Gibbs free energy for main reactions in photocatalytic CO_2_ reduction to CH_4_ for BMO and BMO‐R; Key steps of CO_2_ photoreduction to CO/CH_4_ for c) BMO and d) BMO‐R, in which BMO‐R convert *CO to *OCH. Oxygen of absorbed intermediates, oxygen of BMO/BMO‐R, carbon, bismuth and molybdenum atoms are denoted as balls, respectively red, pink, brown, purple and grey.

To determine the surface species on the surface of BMO and BMO‐R under illumination, in situ DRIFTS were obtained (Figure 4a). The indicative intermediates in CO_2_‐to‐CO conversion *COOH were detected at 1634 cm^−1^.^[25]^ Additionally, the intensity of carbonate species (1577, 1558 and 1304 cm^−1^) was decreased with prolonged illumination time, confirming that the carbonate species is converted to *COOH. Additionally, there were new bands at 1067 cm^−1^ that is attributed to *OCH, and two new bands at 1110 and 1011 cm^−1^ attributed to *OCH_3_.[^9b^, ^24a^] The intensity of these new bands increased highly significantly following illumination, confirming that the corresponding intermediate is accumulated on the surface of BMO‐R. These species are important intermediates to generate CH_4_.^[26]^ However, a different spectrum was obtained for BMO. In Figure S25, the intensity of corresponding carbonate species was much less under illumination. *COOH was detected which confirms CO_2_‐to‐CO.^[27]^ However, there were no bands from *OCH or *OCH_3_. Therefore, it is concluded that the reaction pathways on different samples are:

$${}^{*}\mathrm{CO}{}_{2}\to \mathrm{CO}{}_{2}{}^{-}/\mathrm{HCO}{}_{3}{}^{2-}/\mathrm{CO}{}_{3}{}^{2-}\to {}^{*}\mathrm{COOH}\to {}^{*}\mathrm{CO}\;\mathrm{for}\;\mathrm{BMO}$$

$${}^{*}\mathrm{CO}{}_{2}\to \mathrm{CO}{}_{2}{}^{-}/\mathrm{HCO}{}_{3}{}^{2-}/\mathrm{CO}{}_{3}{}^{2-}\to {}^{*}\mathrm{COOH}\to {}^{*}\mathrm{CO}\to {}^{*}\mathrm{OCH}\to {}^{*}\mathrm{OCH}{}_{2}\to {}^{*}\mathrm{OCH}{}_{3}\;\mathrm{for}\;\mathrm{BMO}\text{-}R$$

The proposed CO_2_ reduction pathway on both samples was assessed via computation of corresponding free energy changes (Δ*G*) for each reaction step, Figure 4b, Figures S26 and S27. The overall energy barrier for BMO‐R is lower than that for BMO, evidencing that the surface condition of BMO‐R significantly facilitates CO_2_ photoreduction. The active site on the regulated surface was distorted, which is in favour of formation of some intermediates thereby reducing the energy barrier for these steps. Because the desorption of CO on BMO is more favourable compared with hydrogenation step, CO is therefore the main product of BMO (Figure 4c). On the contrary, BMO‐R is prone to hydrogenation of *CO (Figure 4d), whereas the *OCH_3_ protonation kinetics barrier is significantly high at 3.02 eV (Figure 4b and S28). The computed adsorption energy for *OCH_3_ on BMO‐R is −1.30 eV. It is speculated that the adsorption configuration of *OCH_3_ is highly stable. Additionally, the high kinetics barrier prevents further transformation, resulting in the significant accumulation of *OCH_3_ on the surface of BMO‐R, and therefore a strong band attributed to *OCH_3_ species from the in situ DRIFTS of BMO‐R.

## Conclusion

A new, active surface‐regulated Bi_2_MoO_6_ nanosheet exhibited high‐performance production of, respectively, 61.5 and 12.4 μmol g^−1^ CO and CH_4_, together with a stability of >20 h of reaction in photocatalytic CO_2_ reduction. The surface was regulated via chemical‐assisted sonication within the nanosheet, and the structure and coordination environment of the surface were confirmed via HAADF‐STEM and EXAFS at the atomic level. Distorted MoO_6−*x*_ is the highly reactive site for light absorption and charge separation. Additionally, this active site significantly promotes CO_2_ activation during pre‐adsorption and photoreduction as confirmed via theoretical computation and in situ spectroscopy. Importantly, hydrogenation of *OCH_3_ for CH_4_ formation was confirmed as the rate‐limiting step. It is concluded the method is generalizable to a wider range of materials to permit regulated surface chemistry for boosted photocatalytic performance. Findings will be of benefit in development of active surface engineering that is applicable to additional photocatalytic systems, including, hydrogen and oxygen evolution, and nitrogen reduction.

## Conflict of interest

The authors declare no conflict of interest.

1

## Supporting information

As a service to our authors and readers, this journal provides supporting information supplied by the authors. Such materials are peer reviewed and may be re‐organized for online delivery, but are not copy‐edited or typeset. Technical support issues arising from supporting information (other than missing files) should be addressed to the authors.

Supporting Information

## Data Availability

The data that support the findings of this study are available from the corresponding author upon reasonable request.
