# Supplementary material for: Facet‐specific Active Surface Regulation of Bi x MOy (M=Mo, V, W) Nanosheets for Boosted Photocatalytic CO2 reduction
Source: Angew Chem Int Ed Engl. 2022 Nov 15;61(50):e202212355. doi: 10.1002/anie.202212355 (PMC10100506; doi:10.1002/anie.202212355)
Supplement: Supplementary file 1 — Supporting Information [file ANIE-61-0-s001.pdf]

Angewandte Chemie
Eine Zeitschrift der Gesellschaft Deutscher Chemiker

Supporting Information

Facet-specific Active Surface Regulation of Bi_xMO_y (M = Mo, V, W) Nanosheets for Boosted Photocatalytic CO_2 reduction

Y. Zhang, X. Zhi, J. R. Harmer, H. Xu, K. Davey, J. Ran, S.-Z. Qiao**

Experimental Procedures

Chemicals and materials

Bismuth nitrate pentahydrate ($Bi(NO_3)_3{\cdot}5H_2O$, Sigma Aldrich), hexadecyltrimethylammonium bromide ($CH_3(CH_2)_{15}N(Br)(CH_3)_3$, CTAB, Sigma Aldrich), sodium molybdat dihydrate ($Na_2MoO_4{\cdot}2H_2O$, Sigma Aldrich), sodium tungstate dihydrate ($Na_2WO_4{\cdot}2H_2O$, Sigma Aldrich), sodium dodecyl benzene sulfonate ($C_{18}H_{29}NaO_3S$, SDBS, Sigma Aldrich), ammonium vanadate (NH_4VO_3, Sigma Aldrich), nitric acid (HNO_3, 70 % Sigma Aldrich), sodium hydroxide (NaOH, Sigma Aldrich), ethanol (C_2H_5OH, ChemSupply), sodium sulfite (Na_2SO_3, Sigma Aldrich). All of the chemicals were Analytical reagents (AR) and used directly without any further purification. Ultra-pure water used throughout all experiments was purified through an Adelab Millipore system. Ultra-high purity Ar (99.999 %) was purchased from BOC Gas, Australia.

Synthesis of Bi_2MoO_6 nanosheets

Bi_2MoO_6 nanosheets were prepared under hydrothermal conditions. 1 mmol of $Na_2MoO_4{\cdot}2H_2O$ and 0.3 g of CTAB were dissolved in 80 mL water and stirred for 30 min. 2 mmol of $Bi(NO_3)_3{\cdot}5H_2O$ was added to the solution and stirred for a further 30 min to obtain a uniform solution. The solution was transferred to a 100 mL Teflon-lined autoclave. The autoclave was transferred into an oven and maintained at 180 ℃ for 16 h. Products were collected and washed with water and ethanol three times. Following drying in a freeze-dryer, the sample was cleaned with plasma cleaner to remove carbon impurities on the surface. Plasma cleaning is detailed below. The resulting samples were labelled as BMO.

Synthesis of $BiVO_4$ nanosheets

$BiVO_4$ nanosheets were prepared by hydrothermal reactions. 2 mmol of $Bi(NO_3)_3{\cdot}5H_2O$ and 1.5 mmol SDBS were dissolved in 20 mL of 4 M HNO_3 solution to form Solution A. 2 mmol of NH_4VO_3 was dissolved in 20 mL of 2 M NaOH solution to form Solution B. B solution was dropped into Solution A and the solution stirred for 30 min. The solution was adjusted to pH ~ 2.5 by 2M NaOH solution and stirred for a further 30 min. It was transferred to 100 mL autoclave and heated under 160 ℃ for 2 h. Products were collected and washed with water and ethanol three times. Following drying in the freeze-dryer, the sample was cleaned with plasma cleaner to remove carbon impurities on the surface. Resulting samples were labelled as BVO.

Synthesis of Bi_2WO_6 nanosheets

Bi_2WO_6 nanosheets were prepared under hydrothermal conditions. 50 mg of CTAB and 1 mmol of $Na_2WO_4{\cdot}2H_2O$ were dissolved in 70 mL water and sonicated for 30 min. 2 mmol of $Bi(NO_3)_3{\cdot}5H_2O$ was added to the solution and sonicated for 30 min to obtain a uniform solution, which was transferred to a

100 mL Teflon-lined autoclave. The autoclave was maintained at 120 ℃ for 24 h. Products were washed with water or ethanol. The products were collected and washed with water and ethanol three times. Following drying in the freeze-dryer, the sample was cleaned with plasma cleaner to remove carbon impurities on the surface. Resulting samples were labelled as BWO.

Plasma cleaning

Following drying of the powders in the freeze-dryer, they were put in the plasma cleaner, vacuumed to 150 mTorr and purged with oxygen (BOC). This was repeated three times until the cleaner was stable at 450 mTorr. The plasma was conducted for 5 min. The powders were washed with water and dried in the freeze-dryer.

Sonication

Sonication was conducted under an ultra-sonic probe. Fifty (50) mg as-prepared samples were dispersed in 100 mL Na_2SO_3 solution (80 mM) for 2 h. The experiment is conducted at 0 ℃ in an ice-water mixture cooling system. The ultrasonic probe was operated for 2 s and stopped for 4 s. The samples were washed with water and dried in the freeze-dryer. Samples following sonication were labelled, respectively, BMO-R, BVO-R and BWO-R.

Material characterizations

X-ray diffraction, XRD data were determined on a powder X-ray diffractometer (Miniflex, Rigaku) using Cu Kα radiation. Raman spectroscopy data were determined using a confocal Raman microscope (Horiba LabRAM HR Evolution) with a 10X objective and a 532 nm laser. Morphology determination was conducted on a Tecnai G2 transmission electron microscopy (TEM). HRTEM, EDX mapping and EELS spectra were obtained on Transmission electron microscopy under STEM mode (FEI Titan Thermis, 200 kV). XPS measurement was on a VGESCALAB 210 XPS spectrometer with Mg Kα source. Binding energies were referenced to the C 1s peak at 284.6 eV. UV visible diffuse reflectance spectra were obtained on a UV-Vis spectrophotometer (UV2600, Shimadzu, Japan). An RF-5301PC spectrofluorophotometer (Shimadzu, Japan) was used to determine steady-state photoluminescence (PL) spectra at room temperature (25 ℃). Transient-state PL decay curves were determined on an FLS1000 fluorescence lifetime spectrophotometer (Edinburgh Instruments, UK). Continuous-wave (CW) X-band (*ca*. 9.385 GHz) electron paramagnetic resonance (EPR) spectra were recorded on a Bruker Elexsys E500 spectrometer equipped with an ElexSys Super High Sensitivity Probehead and He cooling using a cryogen-free cryostat (Bruker waveguide Cryogen-free system with recirculator, WVGD SYS 5K F70H wRCRC 2). The magnetic field was calibrated with a Gauss meter and measurements were carried out using a modulation amplitude of 0.8 mT, a modulation frequency of 100 kHz and a microwave power of

0.5 mW (26 dB of 200 mW, non-saturating condition). Temperature was set to 20 K. Powder samples for EPR were weighted to 0.1 mg and packed in OD 4mm quartz EPR tubes. The height of these samples ranged from 5.9 to 13 mm because of available sample quantity. EPR signal intensity was calibrated by reference to frozen solutions of copper triflate in methanol and included data to cover the variable height of the samples. X-ray absorption was obtained from the XAS beamline of Australian Synchrotron (ANSTO, Melbourne). Data collection was conducted under the transmission mode. XAS raw data were background-substracted, normalized and Fourier-transformed with Athena.

Photocatalytic CO_2 reduction

Photocatalytic performance tests were conducted in a 287 mL reactor sealed with silicone-rubber septa at ambient conditions. A 300 W Xenon lamp was used as the light-source. No light filter was used for BMO, BMO-R, BWO and BWO-R whilst 420 nm filter was used for BVO and BVO-R. In a typical test, 20 mg of photocatalyst was dispersed in 5 mL water and sonicated for 5 min then loaded on a glass-fibre filter to reduced the stack effect and dried under infrared light. Prior to illumination, the reactor was purged with laser-grade CO_2 for 1 h. Product was collected from the reactor h^{-1} *via* syringe and analysed *via* gas chromatograph (GC, 7890B, Agilent). GC was equipped with plot-Q and a 5 Å sieve column (Agilent) in series, TCD and methanizer/FID detectors and UHP Ar (BOC) as carrier-gas. Each test was conducted in triplicate. Blank experiments were conducted under conditions, namely, photocatalysts in ultra-high purity Ar under illumination, photocatalysts in laser-grade CO_2 without illumination, and laser-grade CO_2 under illumination without photocatalysts. Trace/no products were apparent. Importantly, these blank experiments confirmed there were no carbon impurities in the experimental system.

Electrochemical and photoelectrochemical testing

Testing was conducted in a standard, three-electrode system with the as-prepared samples as the working electrode, a Pt wire as the counter electrode, and Ag/AgCl (saturated KCl) as reference electrode. In the identical three-electrode system, EIS measurement was carried out in the range 1 to 2×10^5 Hz with an AC amplitude of 20 mV. 0.5 M Na_2SO_4 was used as electrolyte. Polarization curves were obtained in the three-electrode system. The bias sweep range was –1.5 to –0.8 V *vs.* Ag/AgCl with a step size of 5 mV. 0.5 M Na_2SO_4 was the electrolyte. In the same three-electrode system the TPC response measurement was carried out. A 300 W Xenon light was used as the light source. 0.5 M Na_2SO_4 aqueous solution was the electrolyte. Working electrodes were prepared as follows: 5 mg sample, 960 μL of mixed solvent ($V_{isoproponal}$: V_{water} = 1: 2) with the addition of 40 μL of 5% Nafion. The dispersion was vigorously sonicated for 6 h to form a homogenous ink. A doctor-blade method was used to coat the slurry onto a 2 × 1.5 cm FTO glass electrode.

***In situ* diffuse reflectance infrared spectroscopy (DRIFTS)**

All IR spectra were determined using a Nicolet iS20 spectrometer equipped with an HgCdTe (MCT) detector cooled with liquid nitrogen and a VeeMax III (PIKE technologies) accessory. The *in situ* DRIFTS were determined using a Praying Mantis DRIFTS accessory and a reactor (Harrick Scientific, HVC-DRP). A 300 W Xe lamp (ZhongJiaoJinYuan) is connected to a liquid light guide for irradiation.

Samples were purged with wet CO_2 for 40 min until the sample spectrum was stable. CO_2 adsorption in dark was recorded until it was stable. Spectra under irradiation were recorded as a function of time to determine the dynamics of surface carbon contamination.

Theoretical computation

DFT computations were performed with the Vienna *Ab Initio* Simulation Package (VASP) code.[1] The Perdew-Burke-Ernzerhof (PBE) functional was employed for electron exchange-correlation within the generalized gradient approximation.[2] The projector-augmented wave (PAW) method was used to describe the ionic cores.[3] Geometry optimizations were performed with a 400 eV cut-off energy for plane wave expansion. Ionic relaxations were conducted until all forces were < 0.01 eV·Å^{-1}. A Gaussian smearing was used with 0.2 eV width and a (2 × 2 × 1) Monkhorst-Pack k-point grid was applied. The Tkatchenko-Scheffler method was employed to describe long-range van der Waals interactions.[4]

Kinetic barriers were computed *via* climbing-image nudged elastic band (CI-NEB).[5] Four images were interpolated between initial (IS) and the final state (FS) to determine minimum energy path, and geometry of the transition state (TS). TS was confirmed through frequency analysis to ensure only one imaginary frequency existed, assigned to the unstable mode of minimum energy path.

BMO and BMO-R (010) surfaces were optimized in a 1 × 2 × 1 supercell. A vacuum space of 20 Å was applied to separate the interactions between neighbouring slabs. The computational hydrogen electrode (CHE) model was employed for free energy computations.[6] Free energies for intermediates were obtained from, $G = E + ZPE - TS$. The zero-point energy (ZPE) and entropy correction (TS) were computed from vibration analysis and used to convert electronic energy (E) into free energy (G) at 298.15 K.

Supplementary Figures

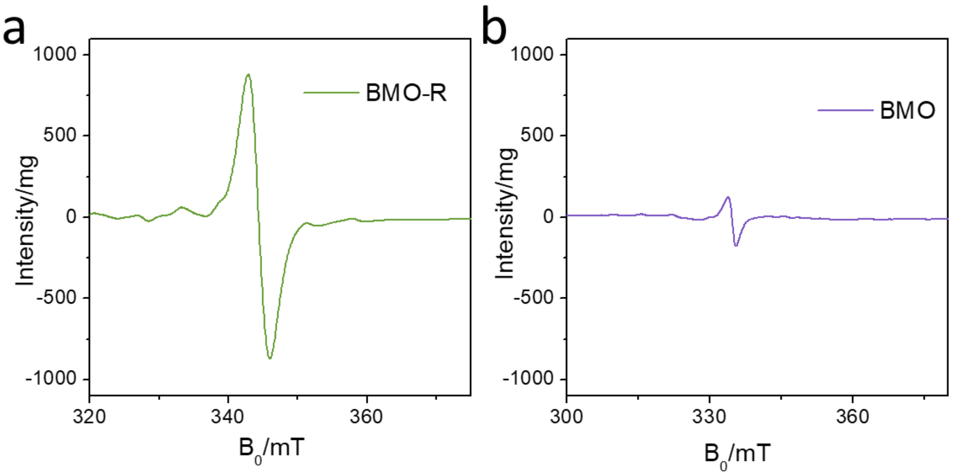

Figure S1. X-band CW EPR spectra for (a) BMO-R and (b) BMO. Double integration of EPR signal gives the number of radicals: 7.5×10^{16} spin/mgram (BMO-R) and 0.05×10^{16} spin/mgram (BMO).

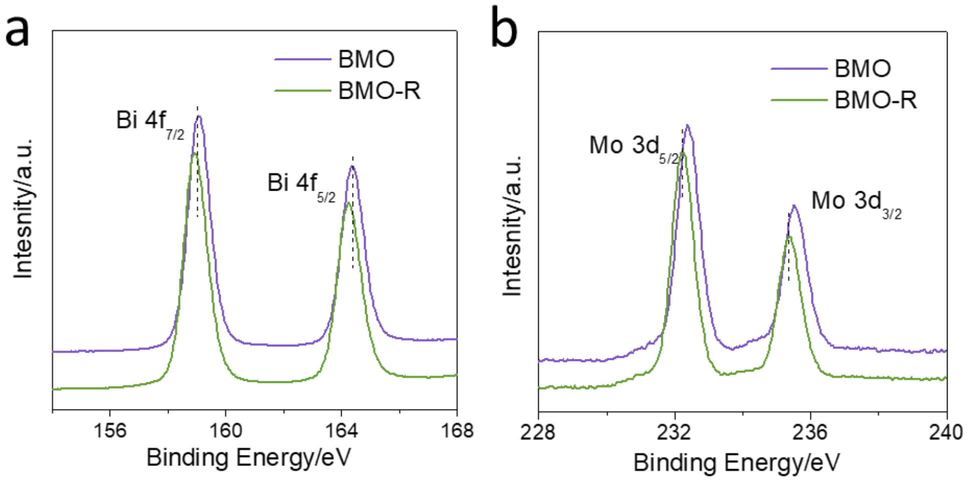

Figure S2. (a) High-resolution XPS spectra for Bi 4f for BMO and BMO-R. (b) High-resolution XPS spectra of Mo 3d for BMO and BMO-R.

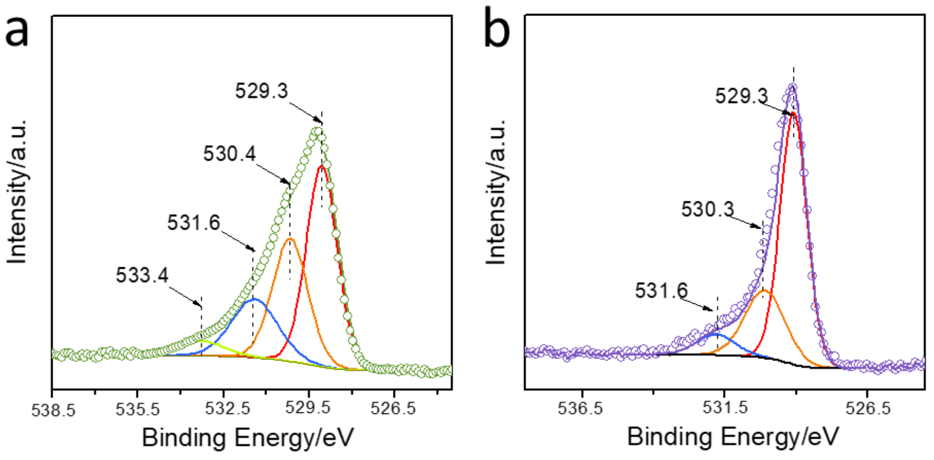

Figure S3. High-resolution XPS spectra for O 1s for (a) BMO-R and (b) BMO. The 529.3 and 530.3 eV peaks are attributed to, respectively, Bi-O and Mo-O bonds.[7] 531.6 eV peak is assigned to oxygen atoms changed by the oxygen vacancy. 533.4 eV peak is from the surface absorbed OH species on BMO-R. It evidences that the regulated surface is more efficient in activating water molecules.

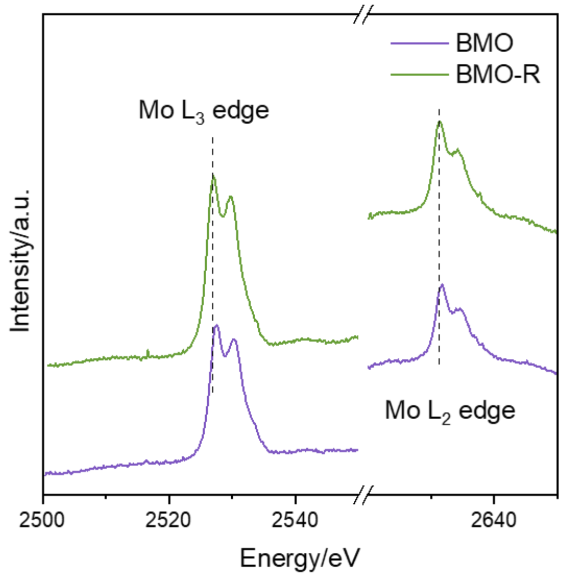

Figure S4. XANES spectra for Mo L edge for BMO and BMO-R.

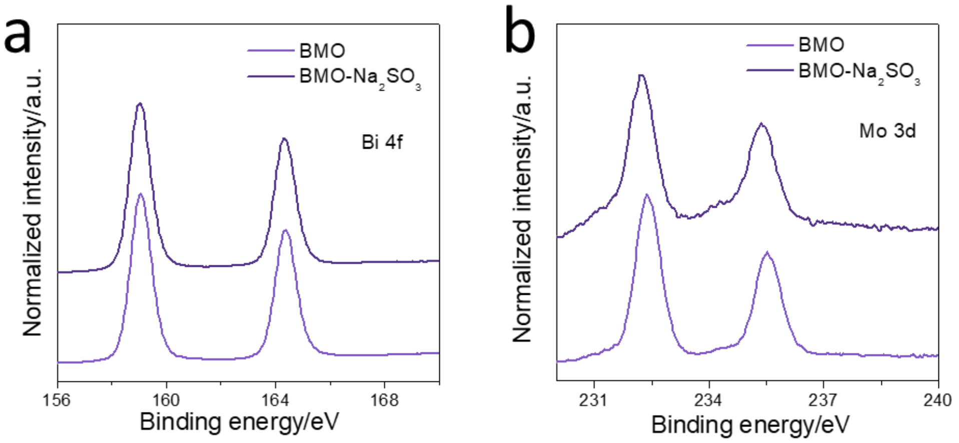

Figure S5. (a) High-resolution XPS spectra for Bi 4f and (b) Mo 3d for BMO and BMO-Na_2SO_3. BMO-Na_2SO_3 was prepared by dispersing BMO in 100 mL Na_2SO_3 solution (80 mM) without sonication for 2 h. It was washed and dried *via* the same procedure as for BMO-R.

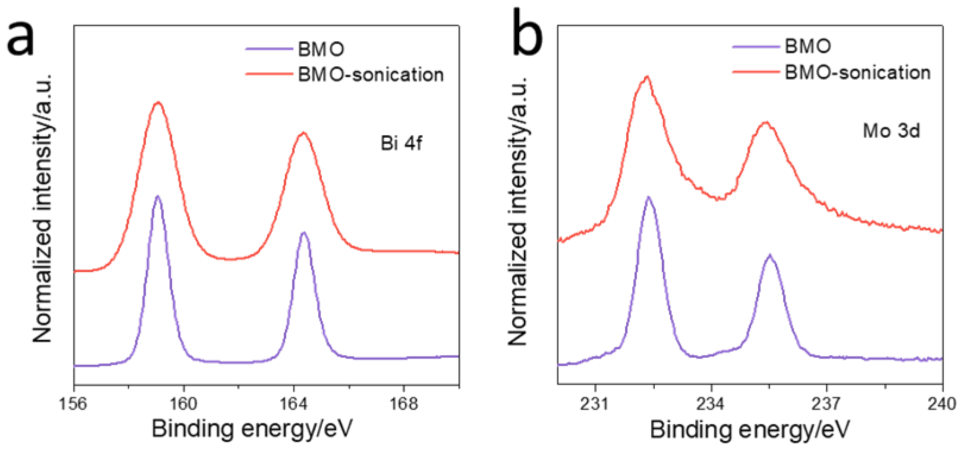

Figure S6. (a) High-resolution XPS spectra for Bi 4f and (b) Mo 3d for BMO, and BMO-sonication. BMO-sonication was in 100 mL of deionized water for 2 h. It was washed and dried *via* the same procedure as for BMO-R.

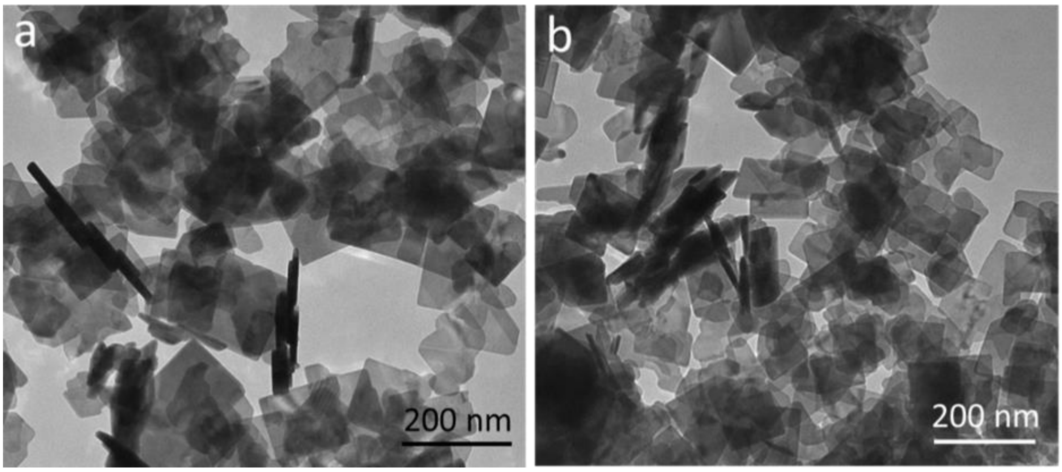

Figure S7. TEM images of (a) BMO and (b) BMO-R.

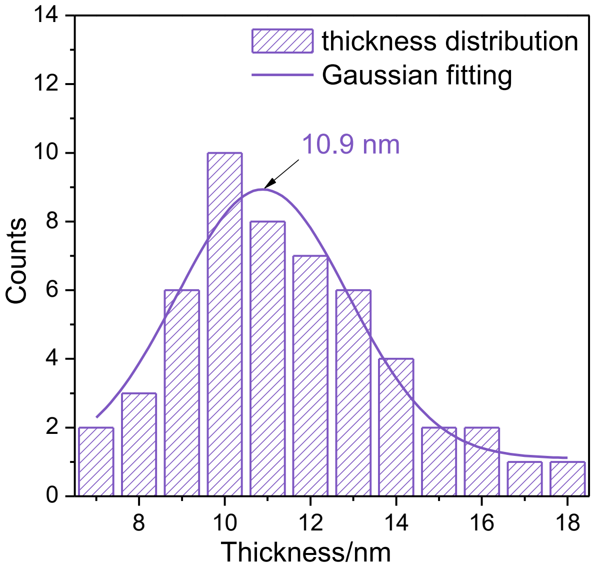

Figure S8. Thickness distribution with Gaussian fitting for BMO.

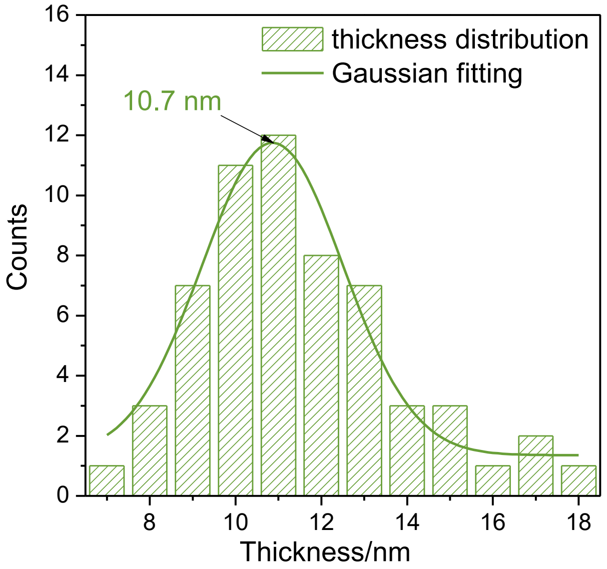

Figure S9. Thickness distribution with Gaussian fitting for BMO-R.

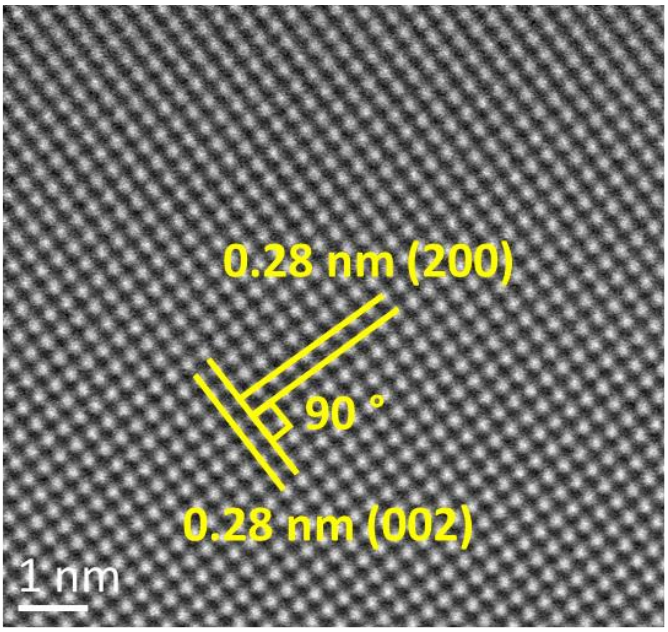

Figure S10. HAADF-STEM images of BMO-R.

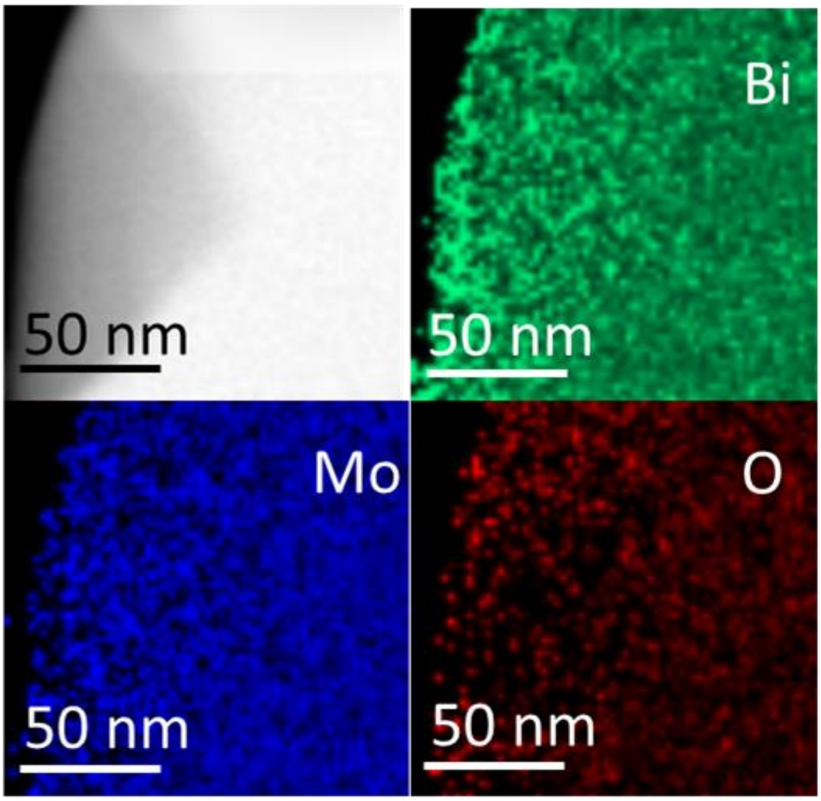

Figure S11. EDX elemental mapping for BMO-R.

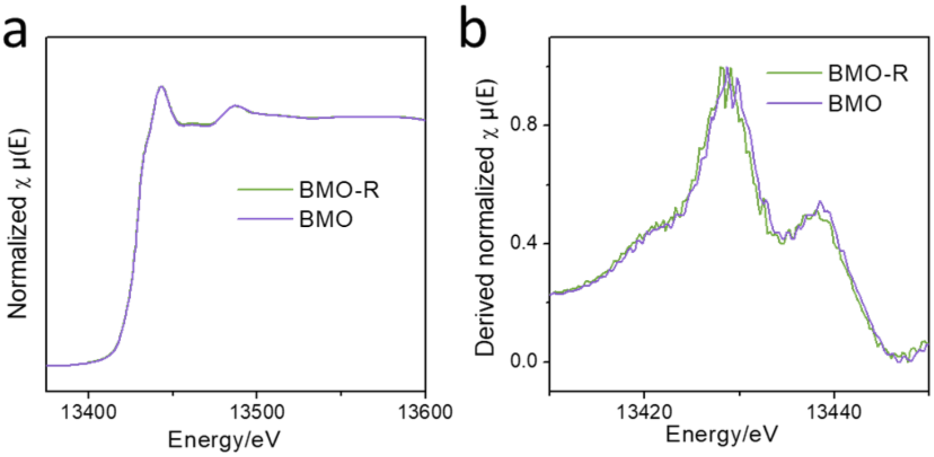

Figure S12. (a) XANES and b) first derivative spectra for Bi L_3 edge for BMO and BMO-R. Peaks in (b) evidence the edge position in (a). There is a slight shift toward lower binding energy for BMO-R, compared with that for BMO, confirming change in chemical state.

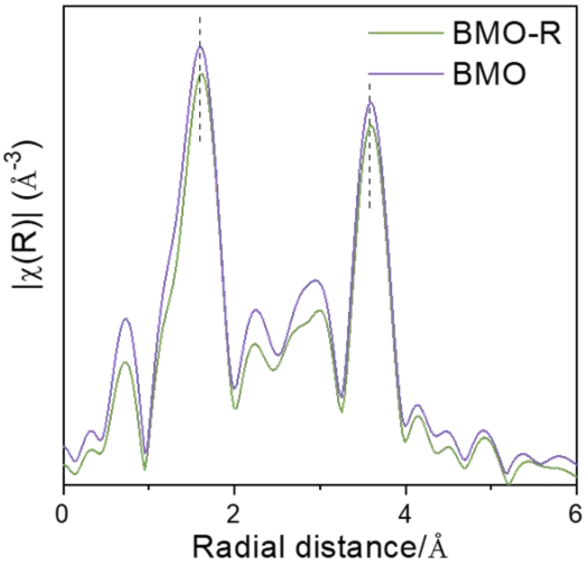

Figure S13. Bi L_3 edge FT-EXAFS for BMO and BMO-R.

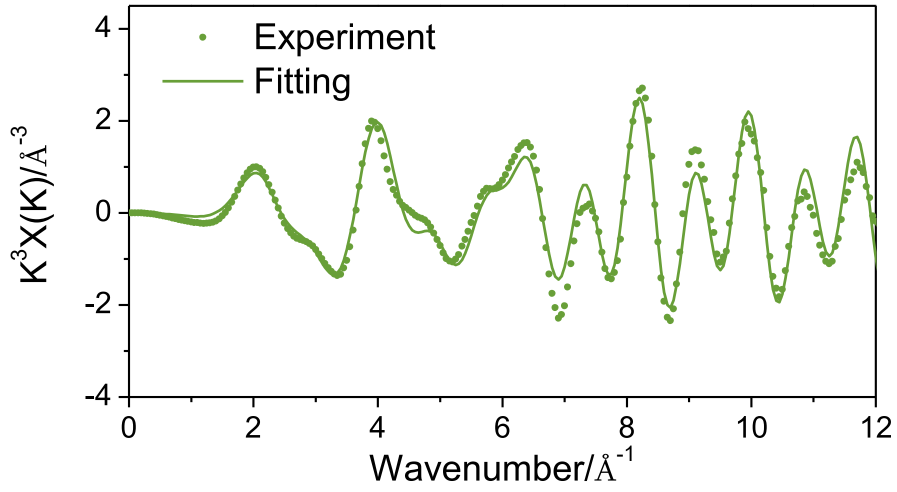

Figure S14. EXAFS fitted curves in k-space for Bi L_3 edge for BMO-R. The dotted line is experimental findings, solid line is fitted spectra.

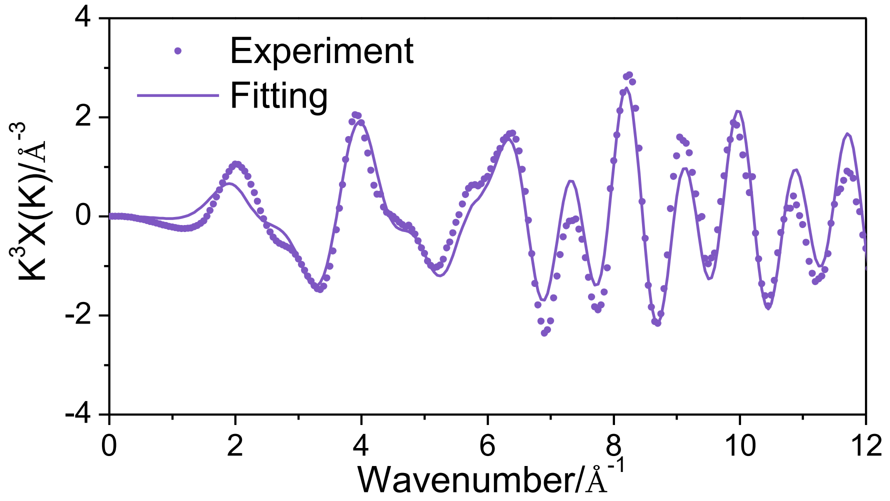

Figure S15. EXAFS fitted curves in k-space for Bi L_3 edge for BMO. The dotted line is experimental findings, solid line is fitted spectra.

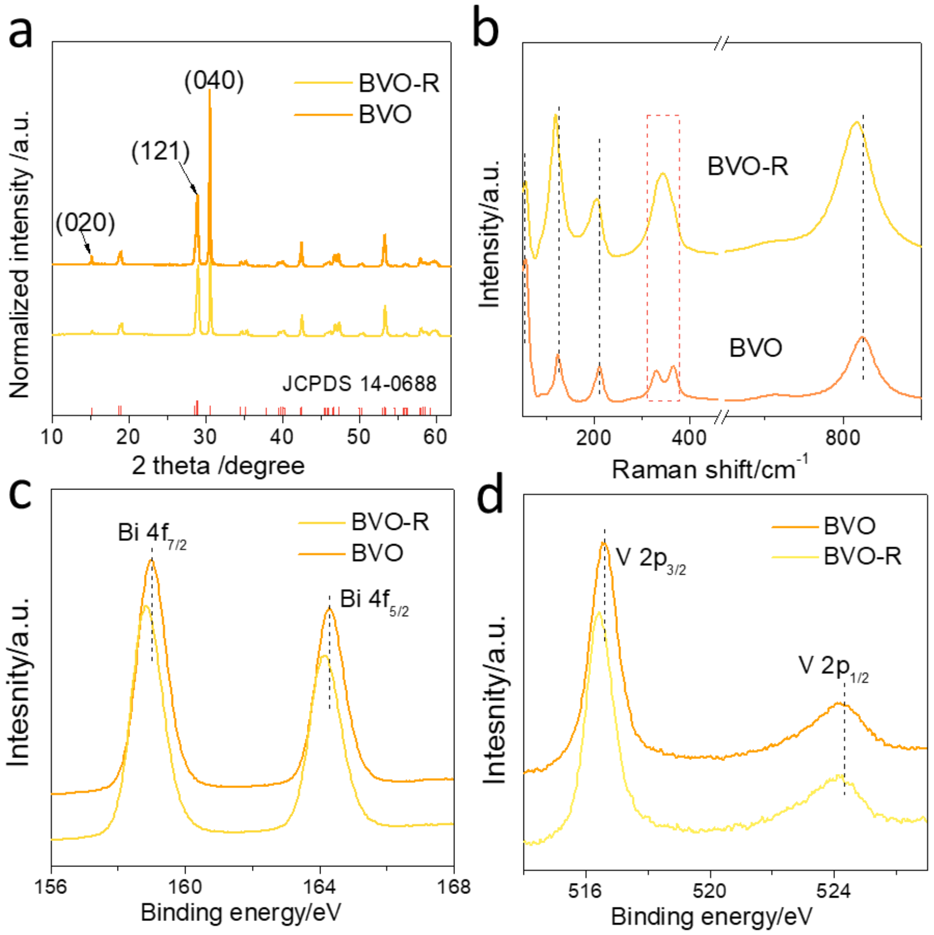

Figure S16. (a) XRD patterns for BVO and BVO-R. All peaks are assigned to JCPDS 14-0688. (b) Raman shift for BVO and BVO-R. High-resolution XPS spectra for (c) Bi 4f and (d) V 2p for BVO and BVO-R.

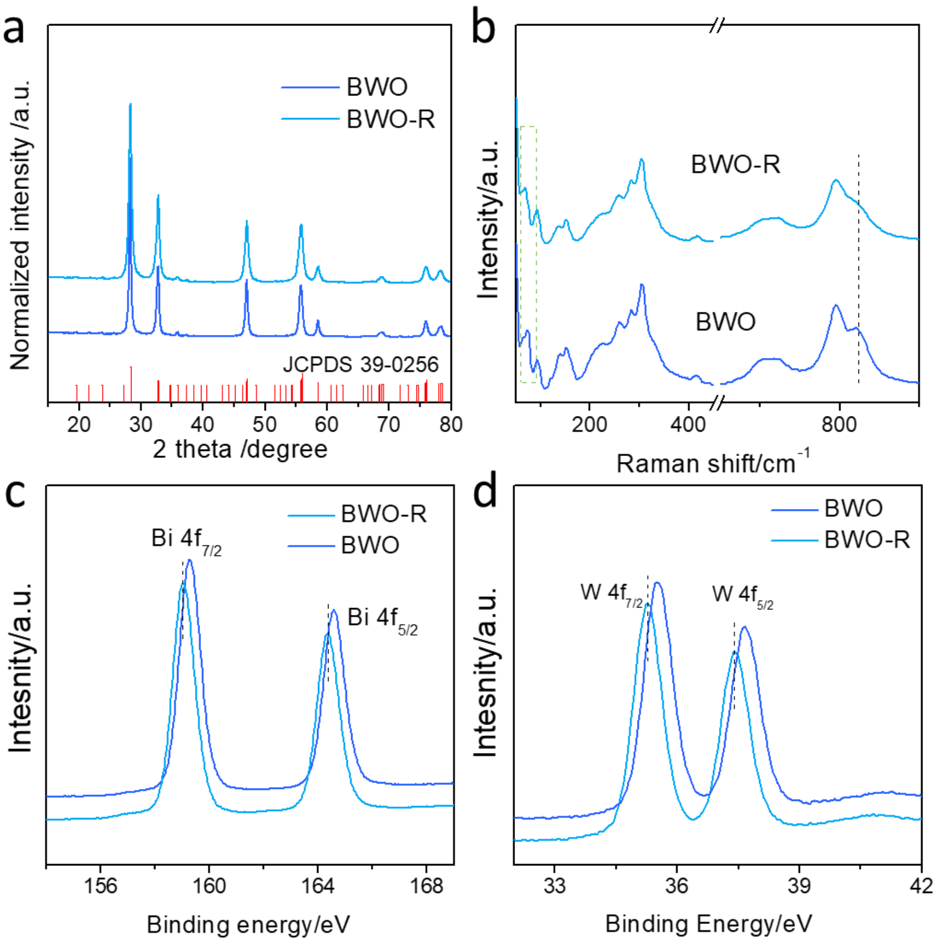

Figure S17. (a) XRD patterns for BWO and BWO-R. All peaks are assigned to JCPDS 39-0256. (b) Raman shift for BWO and BWO-R. High-resolution XPS spectra for (c) Bi 4f and (d) W 4f for BWO and BWO-R.

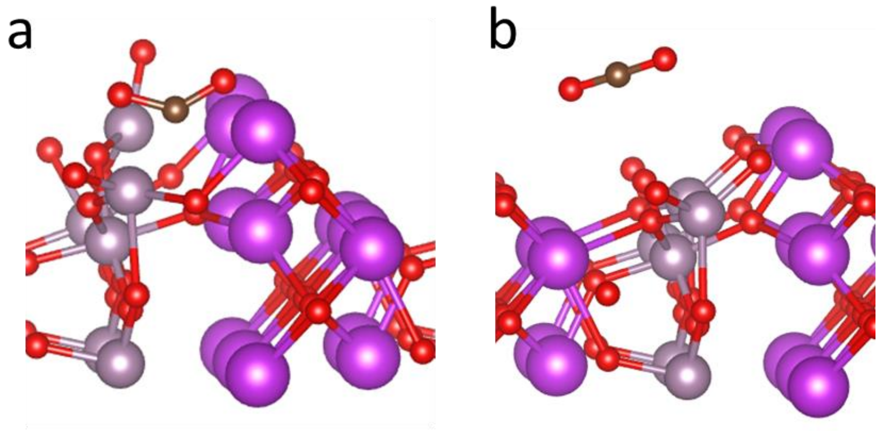

Figure S18. CO_2 adsorption configuration on (a) BMO-R and (b) BMO surface.

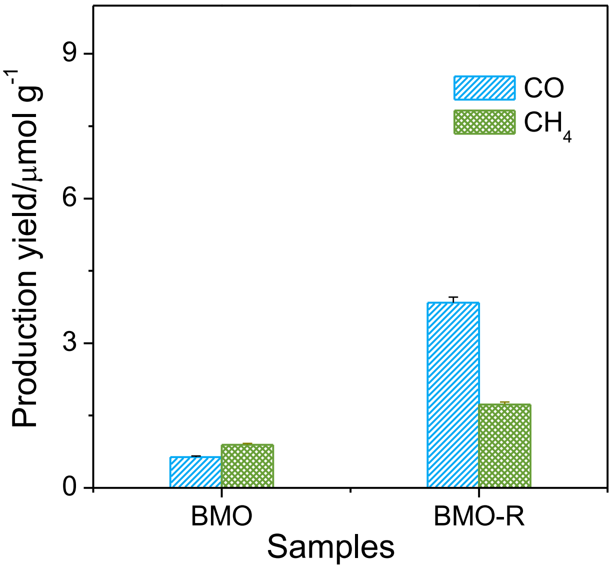

Figure S19. Blank photocatalytic CO_2 reduction test for BMO-R and BMO under illumination with ultra-high-purity Ar for 7 h. No products were detected in other blank experiments.

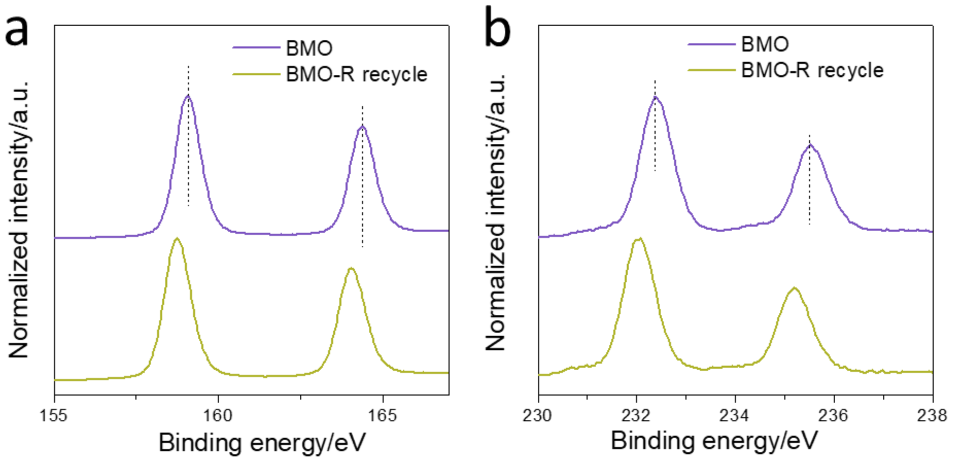

Figure S20. (a) Bi 4f and (b) Mo 3d XPS spectra prior to and following photocatalytic CO_2 reduction. Binding energy shifts remain stable evidencing that the modified surface remains stable during photocatalytic CO_2 reduction testing.

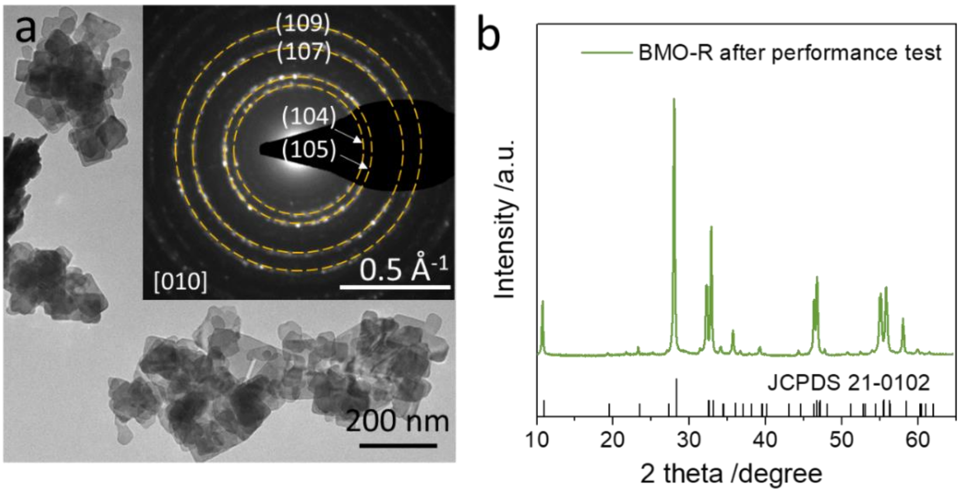

Figure S21. (a) TEM image of BMO-R following photocatalytic CO_2 reduction. Inset is corresponding SAED. The labelled pattern confirms that (010) facets are the dominant exposure facet; (b) XRD patterns for BMO-R following photocatalytic CO_2 reduction performance testing.

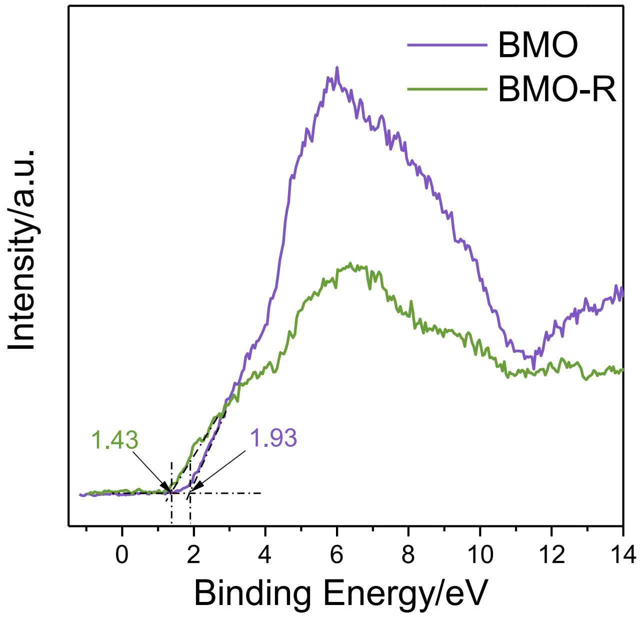

Figure S22. Valence band spectra for BMO and BMO-R samples.

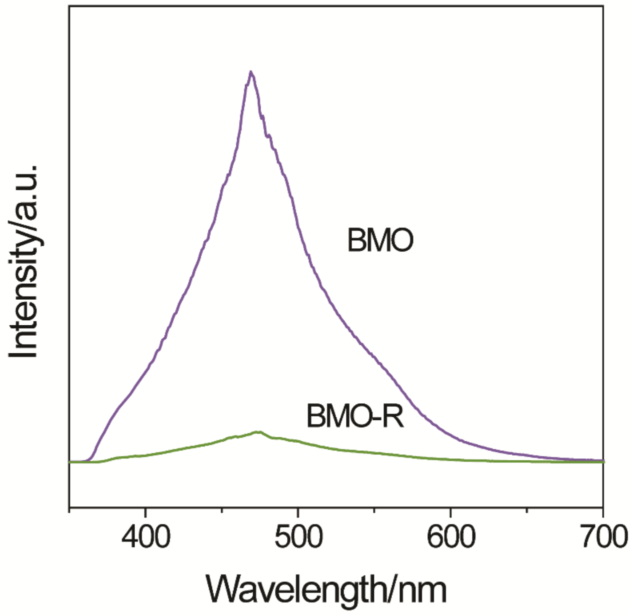

Figure S23. PL spectra for BMO and BMO-R.

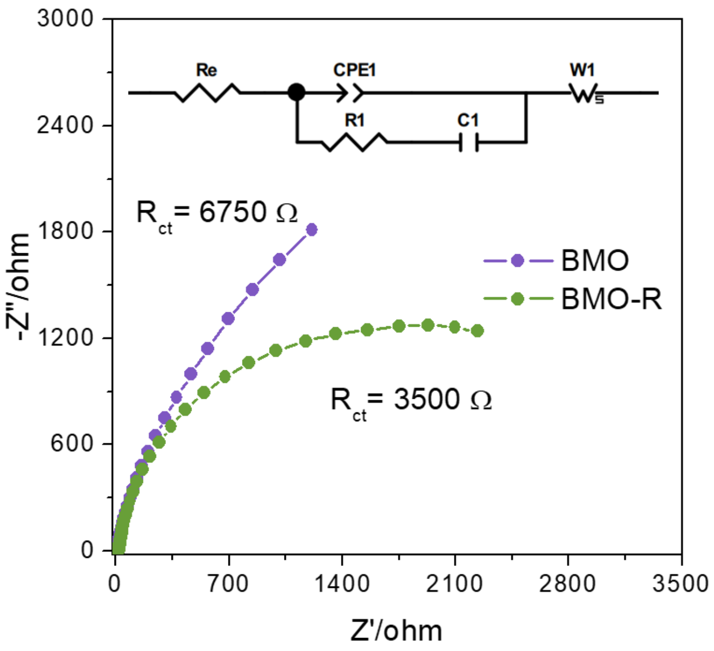

Figure S24. EIS spectra for BMO and BMO-R. Inset is the fitted circuit.

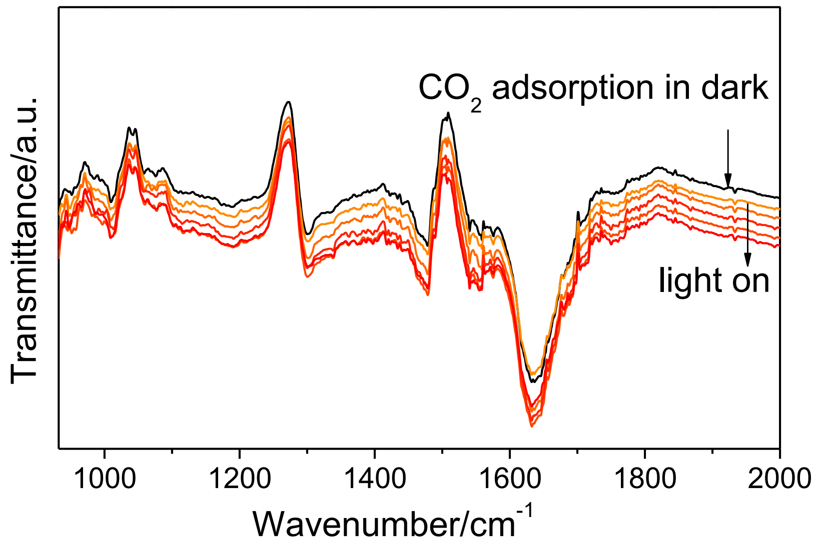

Figure S25. *In situ* DRIFTS for CO_2 photoreduction on BMO. Black-colour line is CO_2 adsorption in dark.

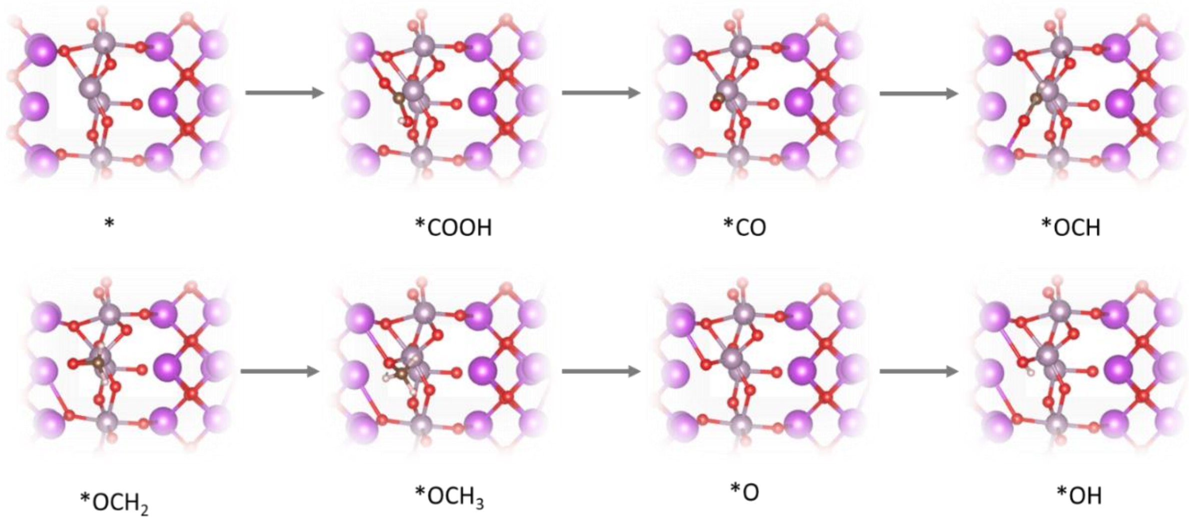

Figure S26. Intermediate configurations for BMO-R along CH_4 pathway. Oxygen, carbon, bismuth, hydrogen and molybdenum atoms are denoted as balls, respectively, red-colour, brown, purple, white and grey.

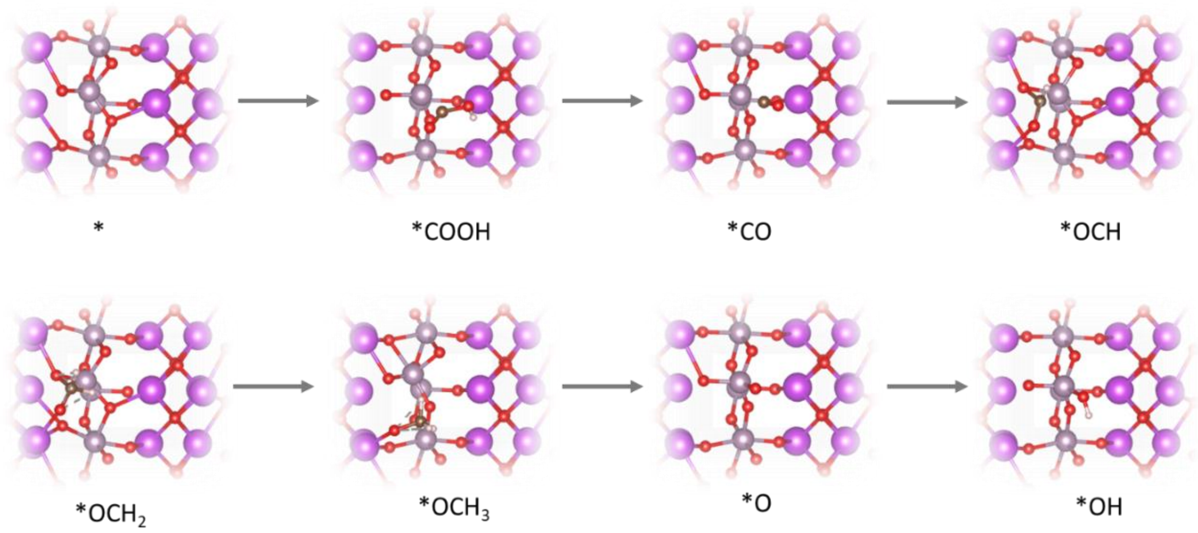

Figure S27. Intermediate configurations for BMO along CH_4 pathway. Oxygen, carbon, bismuth, hydrogen and molybdenum atoms are denoted as balls, respectively, red-colour, brown, purple, white and grey.

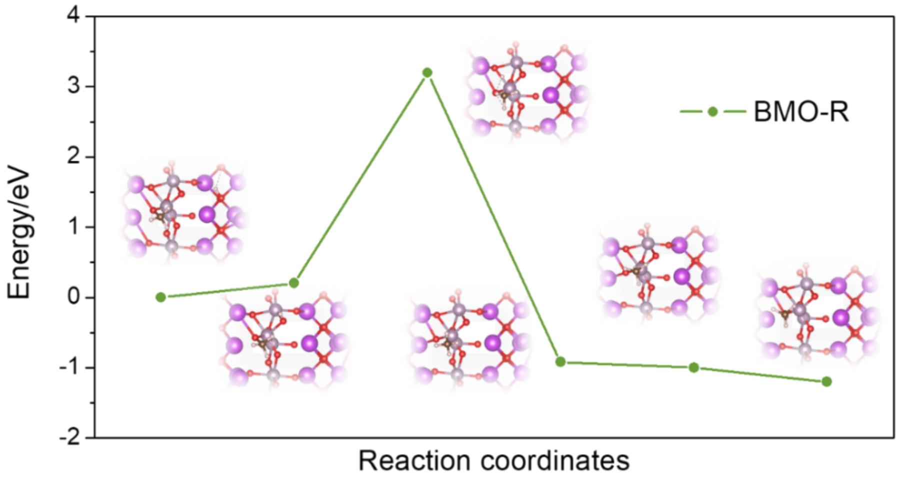

Figure S28. Minimum energy path for BMO-R involved in $*OCH_3 + H \rightarrow *O + CH_4$. Insets are optimized geometry structures for initial, transition and final states. Oxygen, carbon, bismuth, hydrogen and molybdenum atoms are denoted as balls, respectively, red-colour, brown, purple, white and grey.

Supplementary Tables

Table S1. Fitted results for Bi L_3-edge EXAFS data for BMO and BMO-R.

Sample	Path		N (Coordination Number)		R (Å)	*ΔE₀* (eV)	σ² (A²)	R-factor
BMO-R	Bi–O	$Bi–O_1$	5.60	1.94	2.226	3.2435	0.0072	0.03185
		$Bi–O_2$		1.46	2.295			
		$Bi–O_3$		1.99	2.501			
		$Bi–O_4$		0.21	3.657			
	Bi-Bi		6		3.778			
BMO	Bi–O	$Bi–O_1$	5.96	1.87	2.226	5.7059	0.0071	0.02928
		$Bi–O_2$		1.43	2.295			
		$Bi–O_3$		1.37	2.501			
		$Bi–O_4$		1.29	3.657			
	Bi-Bi		6		3.778			

R = the length of Bi–O and Bi–Bi path, *N* = coordination number of Bi atoms corresponding to Bi–O or Bi–Bi path, σ^2 = Debye-Waller factor, and ΔE_0 = inner potential correction.

Table S2. Summary comparison of CO_2 photoreduction performance on Bi-based photocatalysts.

Catalyst	Reaction medium	Light source	Product(s)	Production yield μmol g^{-1} h^{-1}	Ref
Bi_2MoO_6-R	H_2O vapor	Xe lamp	CO, CH_4	8.7, 1.7	This work
Bi_2WO_6-R	H_2O vapor	Xe lamp	CO, CH_4	7.3, 1.02	This work
$BiVO_4$-R	H_2O vapor	Xe lamp	CO, CH_4	2.4, 0.76	This work
Bi_2MoO_6	H_2O	Xe lamp	CO	3.62	[8]
BiOCl-Ov	H_2O	Xe lamp	CO, CH_4	1.01, 0.15	[9]
BiOBr (001)	H_2O vapor	Xe lamp	CO	4.45	[10]
$BiOIO_3$	H_2O vapor	Xe lamp	CO	5.42	[11]
$Bi_4O_5Br_2$	H_2O vapor	Visible light	CO, CH_4	2.73, 2.04	[12]
3D Bi_2MoO_6	H_2O	Xe lamp	CO	41.5	[13]
$ZnFe_2O_4/Bi_2MoO_6$	H_2O	Xe lamp	CO, CH_4	6.5, 2.9	[14]
Ru/Bi_2MoO_6	H_2O	Xe lamp	CO	23.8	[15]
Bi_2WO_6	H_2O vapor	Visible light	CH_4	1.1	[16]
Bi_2WO_6/CODs	H_2O vapor	Visible light	CH_4	0.89	[17]

Bi_2WO_6-V	H_2O vapor	Xe lamp	CO, CH_4	7.7, 0.37	[18]
Bi_2WO_6-C	H_2O vapor	Xe lamp	CO, CH_4	7.12, 0.63	[19]
CdS/$BiVO_4$	H_2O vapor	Xe lamp	CO, CH_4	1.75, 0.39	[20]
$BiVO_4$/EF	H_2O vapor	Visible light	CO, CH_4	1.37, 2.41 μmol cm^{-2} h^{-1}	[21]
Cu/Bi/$BiVO_4$	H_2O vapor	Xe lamp	CO	11.15	[22]

Supporting References

[1] G. Kresse, J. Furthmuller, *Phys. Rev. B: Condens. Matter Mater. Phys.* **1996**, *54*, 11169-11186.

[2] J.P. Perdew, K. Burke, M. Ernzerhof, *Phys. Rev. Lett.* **1996**, *77*, 3865-3868.

[3] G. Kresse, D. Joubert, *Phys. Rev. B: Condens. Matter Mater. Phys.* **1999**, *59*, 1758-1775.

[4] A. Tkatchenko, M. Scheffler, *Phys. Rev. Lett.* **2009**, *102*, 073005.

[5] G. Henkelman, H. Jonsson, *J. Chem. Phys.* **2000**, *113*, 9978–9985.

[6] J.K. Nørskov, J. Rossmeisl, A. Logadottir, L. Lindqvist, J.R. Kitchin, T. Bligaard, H. Jonsson, *J. Phys. Chem. B* **2004**, *108*, 17886-17892.

[7] P.V. Shinde, N.M. Shinde, J.M. Yun, R.S. Mane, K.H. Kim, *ACS Omega* **2019**, *4*, 11093-11102.

[8] J. Di, X. Zhao, C. Lian, M. Ji, J. Xia, J. Xiong, W. Zhou, X. Gao, Y. She, H. Liu, K. P. Loh, S. J. Pennycook, H. Li, Z. Liu, *Nano Energy* **2019**, *61*, 54-59.

[9] L. Zhang, W. Wang, D. Jiang, E. Gao, S. Sun, *Nano Research* **2015**, *3*, 821-831.

[10] D. Wu, L. Ye, H. Y. Yip, P. K. Wong, *Catal. Sci. Technol.*, **2017**, *7*, 265.

[11] F. Chen, H. Huang, L. Ye, T. Zhang Y. Zhang, X. Han, T. Ma, *Adv. Funct. Mater.* **2018**, *28*, 1804284.

[12] L. Ye, X. Jin, C. Liu, C. Ding, H. Xie, K. H. Chu, P. K. Wong, *Appl. Catal. B* **2016**, *187*, 281-290.

[13] X. Zhang, G. Ren, C. Zhang, R. Li, Q. Zhao, C. Fan, *Catal. Lett.* **2020**, *150*, 2510-2516.

[14] Y. Zhou, W. Jiao, Y. Xie, F. He, Y. Ling, Q. Yang, J. Zhao, H. Ye, Y. Hou, *J. Colloid Interface Sci.* **2022**, *608*, 2213-2223.

[15] G. Ren, S. Liu, Z. Li, H. Bai, X. Hu, X. Meng, *Sol. RRL* **2022**, *6*, 2200154.

[16] Y. Zhou, Z. Tian, Z. Zhao, Q. Liu, J. Kou, X. Chen, J. Gao, S. Yan, Z. Zou, *ACS Appl. Mater. Interfaces* **2011**, *3*, 3594–3601.

[17] X. Y. Kong, W. L. Tan, B.-J. Ng, S.-P. Chai, A. R. Mohamed, *Nano Research* **2017**, *5*, 1720-1731.

[18] C. Lu, X. Li, Q. Wu, J. Li, L. Wen, Y. Dai, B. Huang, B. Li, Z. Lou, *ACS Nano* **2021**, *15*, 3529−3539.

[19] Y. Liu, D. Shen, Q. Zhang, Y. Lin, F. Peng, *Appl. Catal. B* **2021**, *283*, 119630.

[20]Z.-H. Wei, Y.-F. Wang, Y.-Y. Li, L. Zhang, H.-C. Yao, Z.-J. Li, *J. CO_2 Util.* **2018**, *28*, 15–25.

[21] S. Yue, L. Chen, M. Zhang, Z. Liu, T. Chen, M. Xie, Z. Cao, W. Han, *Nano-Micro Lett.* **2021**, *14*, 1.

[22] L. Huang, Z. Duan, Y. Song, Q. Li, L. Chen, *ACS Appl. Nano Mater.* **2021**, *4*, 3576–3585.

Author Contributions

Shi-Zhang Qiao conceived the concept and supervised whole project. Yanzhao Zhang is the lead of contribution and charges the methodology, data curation and analysis and writing of original draft. Xing Zhi conducts DFT computation and relative parts writing and reviewing. Jeffrey R. Harmer supports this job in EPR data curation and analysis and relative parts writing and reviewing. Haolan Xu supports the XPS data curation. Kenneth Davey corrects and edits the paper. Shi-Zhang Qiao and Jingrun Ran undertake funding acquisition, supervision, as well as correct and edit the whole paper.
